# Fraisinib: a calixpyrrole derivative reducing A549 cell-derived NSCLC tumor *in vivo* acts as a ligand of the glycine-tRNA synthase, a new molecular target in oncology

**DOI:** 10.3389/fphar.2023.1258108

**Published:** 2024-01-03

**Authors:** Iméne Ben Toumia, Tiziana Bachetti, Leila Chekir-Ghedira, Aldo Profumo, Marco Ponassi, Alessandro Di Domizio, Alberto Izzotti, Salvatore Sciacca, Caterina Puglisi, Stefano Forte, Raffaella Giuffrida, Cristina Colarossi, Danilo Milardi, Giuseppe Grasso, Valeria Lanza, Stefano Fiordoro, Giacomo Drago, Kateryna Tkachenko, Barbara Cardinali, Paolo Romano, Erika Iervasi, Gabriela Coronel Vargas, Paola Barboro, Franz Heinrich Kohnke, Camillo Rosano

**Affiliations:** ^1^ IRCCS Ospedale Policlinico San Martino, Genova, Italy; ^2^ Unit of Bioactive Natural Substances and Biotechnology, Faculty of Dental Medicine of Monastir, University of Monastir, Monastir, Tunisia; ^3^ SPILLOproject, Milano, Italy; ^4^ Department of Experimental Medicine, University of Genova, Genova, Italy; ^5^ Istituto Oncologico del Mediterraneo, Viagrande, Italy; ^6^ Istituto di Cristallografia, Consiglio Nazionale delle Ricerche, Catania, Italy; ^7^ Department of Chemical Sciences, University of Catania, Catania, Italy; ^8^ Dipartimento di Scienze Chimiche, Farmaceutiche ed Ambientali (CHIBIOFARAM), University of Messina, Messina, Italy

**Keywords:** non-small-cell lung cancer, calix[4]pyrroles, proteomics, target discovery, drug discovery, cancer

## Abstract

**Background and purpose:** Lung cancer is the leading cause of death in both men and women, constituting a major public health problem worldwide. Non-small-cell lung cancer accounts for 85%–90% of all lung cancers. We propose a compound that successfully fights tumor growth *in vivo* by targeting the enzyme GARS1.

**Experimental approach:** We present an in-depth investigation of the mechanism through which Fraisinib [meso-(p-acetamidophenyl)-calix(4)pyrrole] affects the human lung adenocarcinoma A549 cell line. In a xenografted model of non-small-cell lung cancer, Fraisinib was found to reduce tumor mass volume without affecting the vital parameters or body weight of mice. Through a computational approach, we uncovered that glycyl-tRNA synthetase is its molecular target. Differential proteomics analysis further confirmed that pathways regulated by Fraisinib are consistent with glycyl-tRNA synthetase inhibition.

**Key results:** Fraisinib displays a strong anti-tumoral potential coupled with limited toxicity in mice. Glycyl-tRNA synthetase has been identified and validated as a protein target of this compound. By inhibiting GARS1, Fraisinib modulates different key biological processes involved in tumoral growth, aggressiveness, and invasiveness.

**Conclusion and implications:** The overall results indicate that Fraisinib is a powerful inhibitor of non-small-cell lung cancer growth by exerting its action on the enzyme GARS1 while displaying marginal toxicity in animal models. Together with the proven ability of this compound to cross the blood–brain barrier, we can assess that Fraisinib can kill two birds with one stone: targeting the primary tumor and its metastases “in one shot.” Taken together, we suggest that inhibiting GARS1 expression and/or GARS1 enzymatic activity may be innovative molecular targets for cancer treatment.

## 1 Introduction

Lung cancer is the leading fatal tumor and in the top five ranked tumors for incidence in the world (Sung et al., 2021), and it has a 5-year survival rate of less than 15% (Wood et al., 2015); overall, non-small-cell lung cancer (NSCLC) accounts for approximately 85%–90% of all cases (Reck et al., 2014). The most effective systemic chemotherapy for NSCLC has been cisplatin (CDDP) or other platinum-based combinations for more than two decades. However, CDDP resistance is a major obstacle to its clinical effectiveness (Sève and Dumontet, 2005). Other chemotherapies include paclitaxel (Taxol), docetaxel (Taxotere), gemcitabine, vinorelbine, etoposide (VP-16), and pemetrexed (Derks et al., 2017), but all of these cause severe adverse effects (AEs), including nausea, fatigue, ulcers, and hair loss. In recent years, treatment strategies have changed with the introduction of specific targeted therapy and immunotherapy (Saar et al., 2023). Immunotherapies are “state-of-the-art” therapies that use the body’s natural defenses to fight the tumor by stimulating/inhibiting different immune system pathways. Some of the most adopted strategies are based on drugs that block the PD-1 pathway and drugs that block the CTLA-4 pathway. In this category of therapies, we can also include therapeutic cancer vaccines and chimeric antigen receptor (CAR) T-cell therapy. Along with chemotherapeutics, immunotherapies can induce side effects that are known as “immune-related adverse effects.” In addition, immunotherapies are often combined with classical chemotherapeutics (Saar et al., 2023).

In general, stage I and stage II NSCLC are treated with surgery, while patients with stage IV NSCLC typically do not undergo surgery or radiation therapy but are instead treated with conventional chemotherapeutics, targeted therapies, or immunotherapy. Palliative care is also important to help relieve symptoms and side effects. Patients with stage III NSCLC may undergo surgery or not, depending on their clinical state and on the size and location of the tumor and the lymph nodes that are involved. For those patients whose tumors cannot be removed with surgery, ASCO recommends radiotherapy using a platinum-based chemotherapy combination.

Nowadays, targeted therapy is considered the standard first-line treatment for epidermal growth factor receptor (EGFR)- or anaplastic lymphoma kinase (ALK)-positive patients. However, only 15%–50% of patients with NSCLC exhibit an activating EGFR mutation (Reck and Rabe, 2017), and ALK translocations occur in just 2%–20% of patients (Blackhall et al., 2014; Steuer and Ramalingam, 2014; Wu et al., 2016). Despite this, CDDP remains the standard first-line chemotherapy for advanced NSCLC (Wang et al., 2004).

**FIGURE 1:**
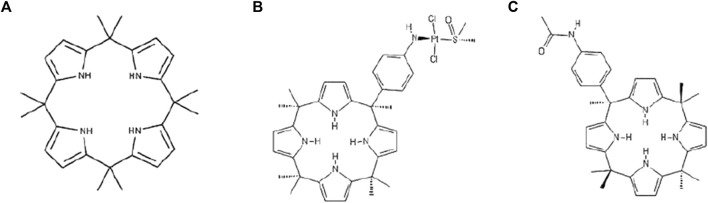
Formula of **(A)** calix[4]pyrrole (C4PY), **(B)** a *meso*-p-nitroaniline-calix[4]pyrrole derivative trans-coordinated to a Pt(II) center, and **(C)** *meso*-(p-acetamidophenyl)-calix[4]pyrrole (Fraisinib).

Calix (Sève and Dumontet, 2005) pyrroles (C4PYs) are members of a very interesting chemical family of macrocycles. They consist of four pyrrole units linked by quaternary carbon atoms at their 2,5 positions, thus forming a ring structure (Figure 1A). These compounds gained considerable interest after the discovery of their ability to bind anions (Gale et al., 1996; Gale et al., 2001), act as ditopic (ion-pair) receptors (Custelcean et al., 2005), and form complexes and “host” neutral molecules containing hydrogen bond acceptor moieties that can interact with NH units of the pyrroles (Allen et al., 1996; Gale, 2011). The ability of C4PYs to bind anionic compounds inspired us to use these macrocycles as “vectors” for the delivery of a trans-Pt moiety to the DNA (Cafeo et al., 2013). Initially, a *meso*-p-nitroaniline-calix (Sève and Dumontet, 2005) pyrrole derivative trans-coordinated to a Pt(II) center was prepared (Figure 1B) that showed its potential as an anticancer drug. This work paved the way for the use of C4PYs in medicinal chemistry. Following this experience, an in-depth investigation into C4PYs was conducted, both as potential vectors for drug delivery (Cafeo et al., 2014; Cafeo et al., 2015) and as innovative drugs (Lappano et al., 2015; Geretto et al., 2018). This led to our discovery that *meso*-(p-acetamidophenyl)-calix (Sève and Dumontet, 2005) pyrrole (Figure 1C), named Fraisinib, is effective against several tumor cell lines (Geretto et al., 2018), particularly on NSCLC. Initially, we were convinced that the main molecular mechanism of cytotoxicity of Fraisinib was due to its capability to form adducts to the DNA, as these were found experimentally. However, the selectivity toward a limited number of tumors, also confirmed by further studies, could not be explained by the generic formation of DNA adducts. The molecular mechanisms underlying Fraisinib’s effects were hypothesized to be mediated by its binding to a specific protein, and this prompted us to investigate Fraisinib targets on a proteome-wide scale using SPILLO-PBSS software (Di Domizio et al., 2014) as this approach had already been successfully used in other similar projects (Giatti et al., 2021; Malacrida et al., 2022). In contrast with traditional structure-based approaches (e.g., molecular docking simulations), this software is more likely to identify targets and off-targets of any small molecule due to its ability to recognize protein binding sites even when they are hidden or highly distorted (cryptic binding sites). This analysis led us to the identification of the protein glycyl-tRNA synthetase 1 (GARS1) as the main target of Fraisinib. GARS1 is an enzyme that is essential in protein synthesis by charging tRNA with glycine amino acid. There is considerable experimental evidence correlating its overexpression with poor prognosis in patients with breast, lung, renal, and liver cancer (Sung et al., 2022). The role of GARS1 in cancer progression was recently linked to the additional functionality of this protein in regulating neddylation, a post-translational modification involved in cell cycle regulation and proliferation (Mo et al., 2016). Furthermore, unlike other tRNA synthases, GARS1 uses direct ATP condensation to synthesize the metabolite diadenosine tetraphosphate (Ap4A) by a unique amino acid-independent mechanism. Ap4A is a secondary signaling molecule that is thought to act as an “alarmone,” signaling cellular stress to evoke an intracellular response (Götz et al., 2019).

This work demonstrates, through a functional test, that Fraisinib suppresses the synthesis of Ap4A by GARS1 and, consequently, that GARS1 is indeed the protein target for this lead compound, as predicted by SPILLO-PBSS. Data obtained by differential mass spectrometry experiments were further explored using computational system biology and bioinformatics to shed more light on the molecular pathways that are influenced by the action of Fraisinib on the human NSCLC cell line A549.

## 2 Materials and methods

### 2.1 Chemistry

Calixpyrrole-derivative Fraisinib and the related molecules shown in Supplementary Figure S1A were prepared as previously reported by Geretto et al. (2018), and their purity was confirmed by both chromatographic and spectroscopic analyses. All compounds used were >95% pure as per HPLC.

### 2.2 A549 cell culture

The human lung adenocarcinoma A549 cell line was purchased from ATCC and cultured in DMEM (Sigma-Aldrich, Milan, Italy) supplemented with 10% fetal calf serum (Euroclone, Milan, Italy), 2 mM L-glutamine (Euroclone, Milan, Italy), and 1% penicillin–streptomycin (Euroclone, Milan, Italy) at 37°C in a 5% CO_2_ incubator. In each experiment, they were used at 70%–80% confluency. A549 cells were chosen because they were well documented to be suitable for studies on lung tumors and drug discovery (Małgorzata Juszczak et al., 2016).

### 2.3 Fraisinib solution for cell treatment and multicaspase assay

**FIGURE 2:**
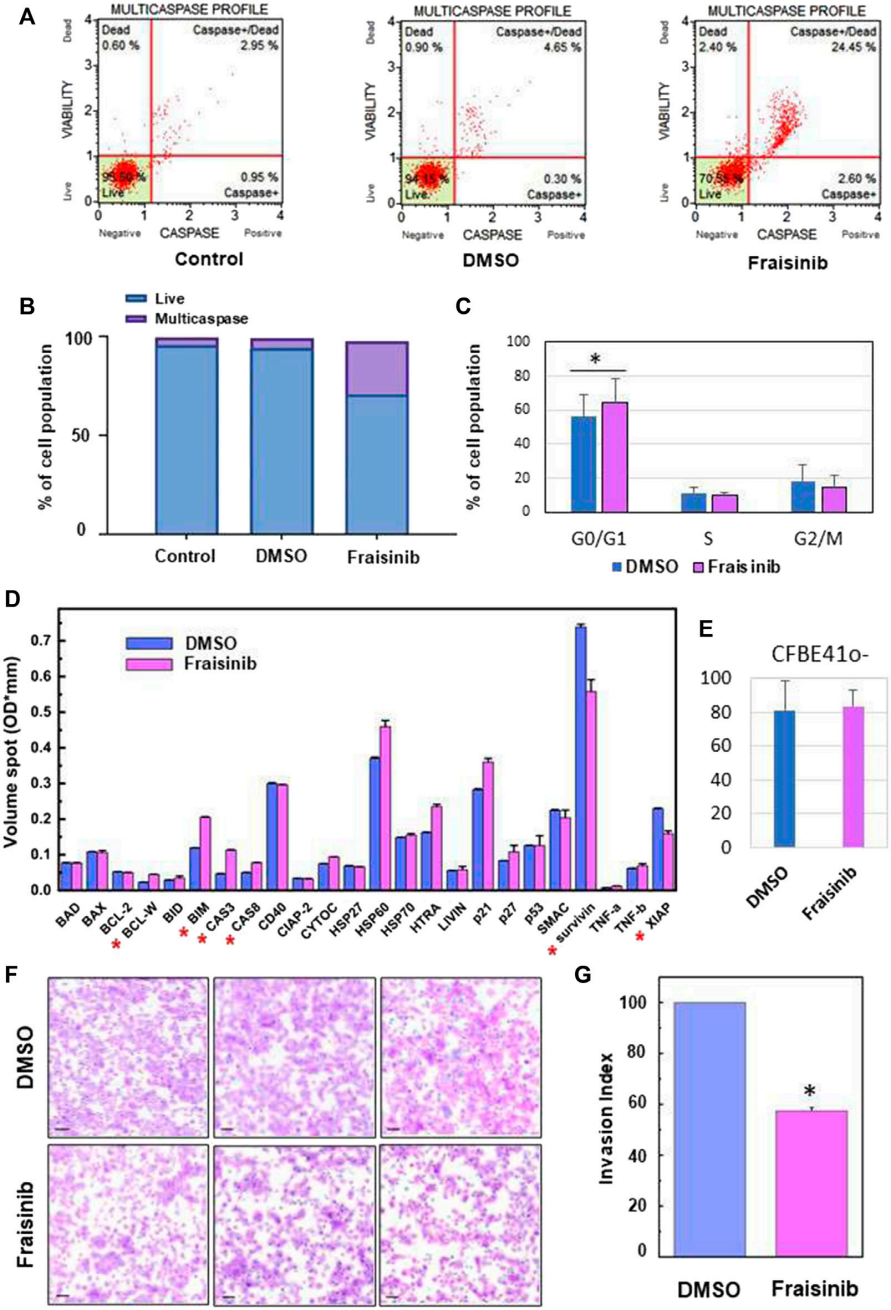
Fraisinib treatment induces caspase cascade activation, cell cycle arrests at the G0/G1 phase, and apoptosis activation and invasiveness inhibition in A549 cells. **(A)** FACS-based activation assays of the multicaspase complex in A549 cells. Profiles were determined in untreated cells (control) and after 48 h treatment with DMSO or 10 μM Fraisinib. **(B)** Bar charts depict the percentage of live cells and multicaspase enzyme activation. **(C)** Relative fractions of cells in G0/G1, S, and G2/M stages determined for cells exposed to DMSO or 10 μM Fraisinib. Statistical difference between DMSO- and Fraisinib-treated cells in G0/G1 was calculated by paired Student’s t-test (asterisk: *p*-value = 0.01). **(D)** Comparison of the expression level of apoptotic proteins extracted from DMSO- and Fraisinib-treated A549 cells. Results are the mean ± SD of two independent wells within the array; asterisks indicate the protein level ratio between treated vs. untreated cells higher than 20%. **(E)** Bar diagram showing the percentage of damaged cells revealed by c.Live Tox reagent 250 nM (Cytena) in DMSO- and Fraisinib-treated cells after 48 h treatments. Values are the mean ± SD of three experiments performed in quadruplicate. **(F)** Images representing cell migration in three independent experiments performed to evaluate invasion inhibition in A549 cells with added DMSO (top images) or exposed to Fraisinib (bottom images). The bars correspond to 100 μm. **(G)** Histogram reporting the mean value ± SD of the migration index of the three independent experiments (asterisks: *p*-value< 0.001).

A stock solution of Fraisinib in DMSO 10 mM was diluted 1:100 with a culture medium. 20 μL of this solution was added to the wells containing the cells in 180 µL of culture medium to obtain a final concentration of 10 µM for Fraisinib and 0.1% DMSO. A mixture of the culture medium containing the same amount of DMSO but without Fraisinib was used for the sham test indicated as “DMSO.” Therefore, the concentration of DMSO in the cell wells was identical for Fraisinib-treated and DMSO-treated cells. An additional set of cells were not treated with any solution, and they are referred to in the main text as control (see Figure 2A).

After 48 h treatment, the Muse™ MultiCaspase Assay Kit (Millipore Merck, Vimodrone MI, Italy) was used for the detection of multiple caspase activation (caspase-1, -3, -4, -5, -6, -7, -8, and -9), following the manufacturer’s instructions. The percentage of cells with multicaspase activity was then examined using a Muse™ Cell Analyzer (Millipore Merck, Vimodrone MI, Italy) flow cytometer. The signal intensity for each antigen-specific antibody spot is proportional to the relative concentration of the antigen, and thus of the protein, in the sample.

### 2.4 Cell cycle arrest analysis

Cell cycle analysis was performed using nuclear DNA intercalating stain propidium iodide (PI) provided in the Muse Cell Cycle Kit (Millipore). Fraisinib cell cycle arrest analysis was carried out as previously described by Parodi et al. (2016). Cells were at 80% confluency for 48 h with Fraisinib compared to the controls; after fixation for 3 h in 70% EtOH at −20°C and washing in PBS, cells were added with the Muse Cell Cycle reagent and incubated for 30′ at RT. The cell cycle was then measured and determined following the manufacturer’s instructions using the Muse Cell Analyzer (Millipore Merck, Vimodrone MI, Italy).

### 2.5 Human apoptotic protein array

To detect simultaneously the relative levels of expression of many apoptosis-related proteins in a single sample, their quantification was carried out using a Human Apoptosis Antibody Array Kit (RayBiotech, GA, United States of America) following the manufacturer’s instructions. A549 cells were seeded (10^5^ cells/mL) and treated with Fraisinib 10 μM; cells added with only DMSO were used as the negative control. After 48 h of treatment, cells were harvested, spun down at 1,500 rpm at 4°C for 5 min, and washed twice with ice-cold PBS. Centrifugation was carried out again at 1,500 rpm at 4°C for 5 min, and the supernatant was discarded. Cell proteins were extracted, and approximately 500 μg of proteins from each sample were incubated with the human apoptosis array overnight. Chemiluminescence detections were carried out by scanning the membrane on an Odyssey Fc Imaging System (LI-COR, United States of America). Results are represented as the medium ±SD of results from two independent wells within the membrane.

### 2.6 Cell invasion assay

Cell invasion assay was carried out in Matrigel chambers (BD Bio Coat), as previously described by Ferrari et al. (2017). A549 cells were seeded (3 × 104 cells/well) in Matrigel chambers in a serum-free medium containing 0.1% DMSO as the control or 2.3 µM of Fraisinib and the same amount of DMSO. Invasion assay was carried out using a medium supplemented with 20% FBS as the chemoattractant for 18 h. The A549 cells that invaded the lower chamber were fixed with 100% methanol and stained with 1% Toluidine blue in 1% borax. The invasion index was calculated as the ratio between the counts of Fraisinib- and DMSO-treated cells invading the Matrigel chamber, and it was expressed as a percentage.

### 2.7 Fraisinib solution for animal treatment

The injectable solution was prepared by dissolving Fraisinib in DMSO (18.75 mg in 200 µL) and then diluted to 1 mL with olive oil. Thus, 6.66 µL of this solution was required for each gram of mice body weight (BW) to achieve a dosage of 125 mg/kg of mice BW. The solution was used immediately. For the control, a mixture of olive oil and DMSO in the ratio of 9:1 was also prepared.

### 2.8 Mouse strain and toxicity experiments

After an acclimation period, 12 Balb/c mice were randomly divided into two groups of six mice each. One group received subcutaneous injections with 125 mg/kg BW of Fraisinib (one injection every 2 days for 1 month), and the second group was injected with the same volume of solvent mixture (oil/DMSO, 9:1) at the same time intervals.

### 2.9 Hematological and biochemical analysis

After sacrificing the mice, blood aliquots were collected in 300 µL tubes (VACUPLAST) containing ethylenediaminetetraacetic acid (EDTA‐K2) and carefully mixed by inversion in a homogenizer (Electra—Homolaby 22T) for hematological tests that were performed in an automated hematology analyzer (Sysmex XE-5000 hematology analyzer, Sysmex, Kobe, Japan) to establish the following parameters: RBC, red blood cells; HB, hemoglobin; HCT, hematocrit; MCH, mean corpuscular hemoglobin; MCHC, mean corpuscular hemoglobin concentration; PLT, platelet count; VGM, mean corpuscular volume; WBC, white blood cells; LYM, lymphocytes; NEU, neutrophils; EOS, eosinophils; MON, monocytes; and BASO, basophiles. For biochemical analysis of serum, aliquots of blood were deposited in tubes (10 × 45 mm, maximum volume 500 µL—VACUPLAST) containing coagulation activators and separator gel. The aliquots were then centrifuged at 2,500 rpm for 5 min (Eppendorf^®^ Minispin^®^ model SPIN 1.000, Hamburg, Germany) to separate the serum. These biological samples were then tested by automated analysis using a commercial Cobas Integra kit (Roche, Boulogne-Billancourt, France) to evaluate the following parameters: creatinine, AST: aspartate aminotransferase, ALT: alanine aminotransferase, uric acid, sodium potassium, chloride, calcium, phosphate, total bilirubin, and phosphatase alkaline.

### 2.10 Tumor growth experiments

Immunodeficient (CD-1 nude) female mice, 12 individuals, were randomly divided into two groups of seven and five mice. The larger group was inoculated with 1 × 10^6^ A549 cells. After 1 week, we started treating the seven-mice group with subcutaneous injections (125 mg/kg of Fraisinib/BW) three times per week. The five-mice group was treated with the same volumes of oil/DMSO (9:1) without Fraisinib (positive control group or DMSO group).

The overall duration of the experiment was 1 month. During the 3-week treatment, tumor growth inhibition was monitored by measuring tumor volumes using the formula V (mm3) = d2 × D/2, where d and D are the shortest and the longest diameters, respectively. At the end of the treatment, tumors were also weighed by using a caliber. Mice B.W. were also monitored.

### 2.11 Protein database preparation

The protein database used for SPILLO-PBSS screening was generated by collecting all protein 3D structures available in the RCSB Protein Data Bank (Berman et al., 2000) (update June 2020) for the organisms *Homo sapiens* (45,872 entries), *Mus musculus* (6,474 entries), and *Rattus norvegicus* (2,970 entries) experimentally solved by either X-ray diffraction or solution NMR, including sequence redundancies, for a total of 55,316 holo- and apo-protein 3D structures. Biological assemblies for proteins showing multimeric structures were then generated in accordance with the BIOMT transformation matrices included in the PDB files. For multi-model PDB files from solution NMR experiments, only the first model was included in the database. No further protein structure refinements were carried out to improve the quality of protein 3D structures in the database.

### 2.12 RBS generation

The reference binding site (RBS) used by SPILLO-PBSS to search the protein database for potential targets of Fraisinib was obtained by molecular modeling techniques and the standard RBS generation protocol described in the SPILLO-PBSS reference paper (Di Domizio et al., 2014). It included 22 amino acid residues directly interacting with Fraisinib without any water-mediated contact.

### 2.13 *In silico* screening and ranking of the protein database

An unbiased and systematic search for Fraisinib potential binding sites (PBSs) within all protein 3D structures included in the database was carried out using SPILLO-PBSS. Calculations were performed using a rotation step of 30° and a grid spacing of 2.0 Å, with the geometric tolerance set to 4.0 Å. SPILLO-PBSS analyzed all proteins in the database, and a ranking of the PBSs for the molecule was obtained for each protein and stored by the program. The proteins were then ranked according to the highest PBS score, representing the highest similarity to the corresponding RBS, obtained from each analyzed protein 3D structure.

### 2.14 Target validation

GARS1 aliquots (0.5 μM) were incubated in the reaction buffer (5 mM HEPES, pH 7.5, 2 mM KCl, 0.5 mM MgCl_2_, and 100 μM DTT) in the presence of 5 mM ATP and different concentrations of inhibitor (from 0.1 to 20 µM) at 25°C. Aliquots of the reaction mixtures (4 μL) were quenched after 30 min by adding 4 μL of a 200 mM EDTA solution and analyzed in a 96-well plate by using a Varioskan (Thermo Fisher Scientific). PPi released during the reaction was measured by the molybdate spectrophotometric assay, as previously reported by Guan et al. (2012). Quenched reaction mixtures were incubated for 10 min with 200 μL of 3 mM ammonium molybdate in 0.6 M HCl (60% CH3CN W/W). The formation of the blue molybdene complex was assayed by measuring the optical absorbance at *λ* = 790 nm. Calibration curves were obtained using standard PPi solutions (0–50 μM). All molecules tested (Supplementary Figure S1) were prepared in DMSO stock solutions to ensure that the final DMSO concentration did not exceed 1% and that the volumes added in each well were the same.

### 2.15 A549 cell treatments and protein extraction

For cell treatments, Fraisinib stock solution 10 mM in DMSO was diluted to the final 10 μM concentration in the culture medium. DMSO (0.1%) was added to control samples at each time point at the same concentration of the diluted drug. After 24, 48, and 72 h, cells were harvested with a cell scraper, washed with PBS added with protease (20 μg/mL leupeptin, 25 μg/mL aprotinin, 10 μg/mL pepstatin, 0.5 mM benzamide) and phosphatase inhibitors (1 mM Na_3_VO_4_), and collected by centrifugation at 4°C at 1,500 rpm for 15 min. The pellet of cells was lysed by RIPA buffer (50 mM Tris-HCl, 150 mM NaCl, 1% Triton X100, 0.1% SDS, and 0.5% sodium deoxycholate, pH 8.0), incubated at 4°C for 60 min, and then, sonicated for 30 s in ice. Following centrifugation at 7,000 rpm for 15 min, the supernatant containing the total proteins was transferred into a new tube; 100 μL DTT 1M:0.9 mL ratio was added to the proteins and then incubated at RT for 30 min. Subsequently, 400 μL 0.5 M iodoacetamide:0.6 mL volume was added followed by incubation at RT for 30 min. Solution C 2X (SDS 20%; DTT 6%) was added at the ratio of 1:1, vortexed, and incubated at 95°C for 5 min to remove lipids and nucleic acids. Proteins were then precipitated with 5 volumes MATF (1 mL methanol:12 mL acetone:1 mL tributyl phosphate), rotated at 4°C for 60 min, and then, centrifuged at 7,000 rpm for 15 min. Pellets were resuspended in 1 mL MATF, centrifuged at 12,000 rpm at 4°C for 15 min, and dried in a SpeedVac. The obtained proteins were resuspended in ammonium bicarbonate (AMBIC) 50 mM to the final concentration of 2 μg/μL, digested with trypsin at 37°C for 1 h, and blocked with acetic acid 50% in a 1:10 ratio. Following agitation for 10 min, tryptic peptides were dried under a vacuum.

### 2.16 Mass spectrometry analysis

Tandem mass analysis of tryptic digests was performed on an UltiMate 3000 nano chromatography system (Thermo Fisher Scientific) equipped with a PepMap RSL C18 column (75 μm × 150 mm; 2 μm particle size) (Thermo Fisher Scientific) at a flow rate of 250 nL/min and a temperature of 60°C. Mobile phase A was 0.1% v/v formic acid in water, and mobile phase B was 80% ACN, 20% H_2_O, and 0.08% v/v formic acid. The following 105 min gradient was selected: 0.0–3.0 min isocratic 2% B; 3.0–7.0 min 7% B; 7.0–65.0 min 30% B; 65.0–78.0 min 45% B; 78.0–83.0 min 80% B; 83.0–85.0 isocratic 80% B; 85.0–85.1 2% B; and finally, 85.1–105.0 isocratic 2% B.

After separation, the flow was sent directly to an EASY-Spray (ESI) source connected to an Exactive Plus Orbitrap Q Mass Spectrometer (both Thermo Fisher Scientific). The software Xcalibur (version 4.1, Thermo Fisher Scientific) was used for operating the UHPLC/HR-MS. MS scans were acquired at a resolution of 70,000 between 200 and 2,000 m/z, with an automatic gain control (AGC) target of 3.0E6 and a maximum injection time (maxIT) of 100 ms. MS/MS spectra were acquired at a resolution of 17,500 and an AGC target of 1.0E5 and a maxIT of 50 ms. A quadrupole isolation window of 2.0 m/z was used, and HCD was performed using 30 normalized collision energy (NCE).

Data from the mass spectrometer in *.raw format was processed with Thermo Fisher Proteome Discoverer^®^ software version 2.4.1.15 using a workflow adapted to LTQ ORBITRAP label-free quantification. The software divides the data analysis into two steps: processing and consensus.

The processing step established the database for PMS identification in MS/MS spectra and concatenated decoy (*Homo sapiens* - sp_canonical v2022-03-02, target FDR strict = 0.01, and target FDR relaxed = 0.05 for proteins, peptides, and PSMs), static modification (Carbamidomethyl/+57.021Da on C), and dynamic modifications (oxidation/+15.995 Da (M); Phospho/+79.966 Da (S, T, Y)), as well as identification engines (MS Amanda 2.0 (Dorfer et al., 2014)) Sequest HT^®^ and tolerances (precursor mass tolerance = 10 ppm and fragment mass tolerance = 0.0 2Da).

In the consensus step, precursor abundance was calculated by intensity, using unique + razor peptides and considering protein for peptide uniqueness. Peptide normalization (based on total peptide amount, scaling on all average), peptide filters (high confidence, minimum length = 6), protein quantification (Unique + Razor, precursor abundance based on intensity), and differential analysis (protein abundance calculation based on summed abundances, pairwise ratio based, and *t*-test background based) were also assessed in this step using PD Precursor Ions Quantifier and IMP-apQuant nodes (https://ms.imp.ac.at/index.php?action=apQuant). The entire workflow is described in Supplementary Table S5. The mass spectrometry proteomics data were deposited in the ProteomeXchange Consortium via the PRIDE (Deutsch et al., 2020; Perez-Riverol et al., 2022) partner repository with the dataset identifier PXD037653 (see “Data availability”).

### 2.17 Functional network construction and enrichment analysis

As explained earlier, identified proteins were used to perform pairwise ratio-based comparisons between untreated and Fraisinib-treated A549 cells at 24, 48, and 72 h. Among the 4,352 high-FDR master proteins identified from all the spectrum files, the 1,077 (24.7%) significantly dysregulated ones (*p*-value <0.05) with an expression change of more than two-fold were collected in the following three groups: differentially expressed protein (DEP) 24 h, DEP 48 h, and DEP 72 h (Supplementary Table S1).

The STRING database was queried using the DEP lists to obtain the protein–protein interaction (PPI) data (medium confidence: 0.4) considering text mining, experiments, databases, co-expression, and co-occurrence as setting, while the GARS1 interactome was also constructed using data from the Protein Interaction Network Analysis (PINA) database. The functional networks reported in Figure 5 and Supplementary Figure S5 were built using Cytoscape software (v3.9.9), and the topological features, reported in Table 1, were evaluated by the Analyze-Network tool.

**TABLE 1:** Differentially expressed proteins and merged-network characteristics.

	24 h		48 h		72 h	
Network feature	DEP network	Merged network	DEP network	Merged network	DEP network	Merged network
Protein building network (node)	426	526	465	563	396	490
Protein excluded from the network	49	36	57	47	58	49
Number of interactions (edge)*	1,171	2,436	1,252	2,531	962	2,012
Average number of neighbors*	5.6	9.3	5.4	9	4.9	8.3
Clustering coefficient*	0.216	0.244	0.203	0.243	0.192	0.241
PPI enrichment *p*-value**	1.8E-11	<1E-16	4.8E-10	<1E-16	1.7E-7	<1E-16

*Provided by the Cytoscape Analyze-Network tool.

**The PPI enrichment *p*-values represent the statistical significance provided by STRING.

Enrichment analysis and the resulting functional group visualization were performed by Cytoscape ClueGO plugin (v.2.5.7) using the following criteria: *p*-value adjusted using the Bonferroni step down <0.02, Gene Ontology (GO) Biological Process and Reactome Pathways, Min GO level 3, Max GO level 6, and kappa score threshold 0.55.

### 2.18 Statistical analysis

To assess the significance of Fraisinib effects on cells, paired Student’s t-test and one-way ANOVA were performed on values obtained from experimental replicates by comparing treated and untreated (DMSO added) samples. To evaluate the statistical significance in animal model experiments, treated and untreated (DMSO added) samples at each time point were compared by using unpaired Student’s t-test.

## 3 Results

### 3.1 Fraisinib induces cell cycle arrest and apoptosis through the caspase cascade activation

To unravel the mechanism underlying the cytotoxicity of Fraisinib, the apoptotic activity was evaluated in A549 cells. The activation of apoptosis initiator and executioner caspases (caspase-1, -3, -4, -5, -6, -7, -8, and -9) was evaluated. As shown in Figures 2A, B, Fraisinib treatment significantly increased the percentage of cells having an activated caspase cascade compared to untreated checks and vehicles.

Due to the very low solubility of Fraisinib in the aqueous medium, it was initially dissolved in a small volume of DMSO and diluted to the required concentration with the culture medium (see Materials and methods). Solutions containing the same amount of DMSO but without Fraisinib were used for the sham tests. In both cases, the final concentration of DMSO in the wells was 0.01%. A second negative control was also used that was not treated with either DMSO or Fraisinib. In Figure 2A, the caspase profiles for these differently treated cells are indicated as Fraisinib, DMSO, and control, respectively.

To further study the mechanisms of action of Fraisinib in A549 cancer cells, a Muse™ MultiCaspase Assay Kit (Millipore Merck, Vimodrone MI, Italy) was used to examine the cell cycle arrest ability of Fraisinib (at 10 µM concentration in normoxic conditions). Fraisinib efficiently and significantly increased a percentage of the cell population in the G0/G1 phase compared to the DMSO-treated cells (Figure 2C). These combined results clearly illustrate the capacity of Fraisinib to control cell cycle progression via caspase cascade (Hashimoto et al., 2011). To evaluate the expression levels of several proteins involved in apoptosis, a semi-quantitative analysis of apoptotic proteins in A549 cells treated with 10 μM Fraisinib for 48 h was carried out using a human apoptotic protein array.

In particular, the difference in signal intensities relative to each protein between Fraisinib- and DMSO-treated cells (Supplementary Figures S1A, 1B) was evaluated to determine the differences in relative protein expression across the full array of human apoptotic proteins (Figure 2D).

After evaluating protein levels in treated vs. DMSO cells, only proteins that showed at least 20% up- or downregulation after Fraisinib treatment were considered possible players in the apoptotic process regulated by Fraisinib. Among these, the upregulated BCL-W, BIM, caspase-3, and Caspase-8 and the downregulated XIAP and survivin suggest that the apoptotic pathway is activated by the drug through both the induction of pro-apoptotic proteins and the reduction of proteins acting as apoptosis inhibitors.

Last but not least, Fraisinib treatment did not show toxic effects on the normal lung cells CFBE41o (Figure 2E).

### 3.2 Fraisinib inhibits the invasive ability of A549 cells

We evaluated the inhibitory effect of Fraisinib on the invasive ability of A549 cells using a Matrigel invasion assay. In line with previous results (Geretto et al., 2018; Ben Toumia et al., 2022), this test was conducted using the IC80 (2.3 µM). Compared to cells treated with DMSO, Fraisinib displayed a marked inhibitory effect against A549 cell invasiveness, decreasing the invasion rate by 57.4% (Figures 2F, G). This *in vitro* assay suggests that Fraisinib may inhibit the metastatic potential of A549 cells.

### 3.3 Toxicity evaluation

In a previous study, we demonstrated that Fraisinib showed no cytotoxic effect on lymphocytes (peripheral blood mononuclear cells, PBMCs) in the 0.1–300 µM concentration range. *In vivo* studies in mice showed no alterations in behavior or change in BW after treatment for 72 h at a dose of 50 mg/kg of BW, and no significant changes were detected in transaminases and creatinine serum levels (Ben Toumia et al., 2022).

**FIGURE 3:**
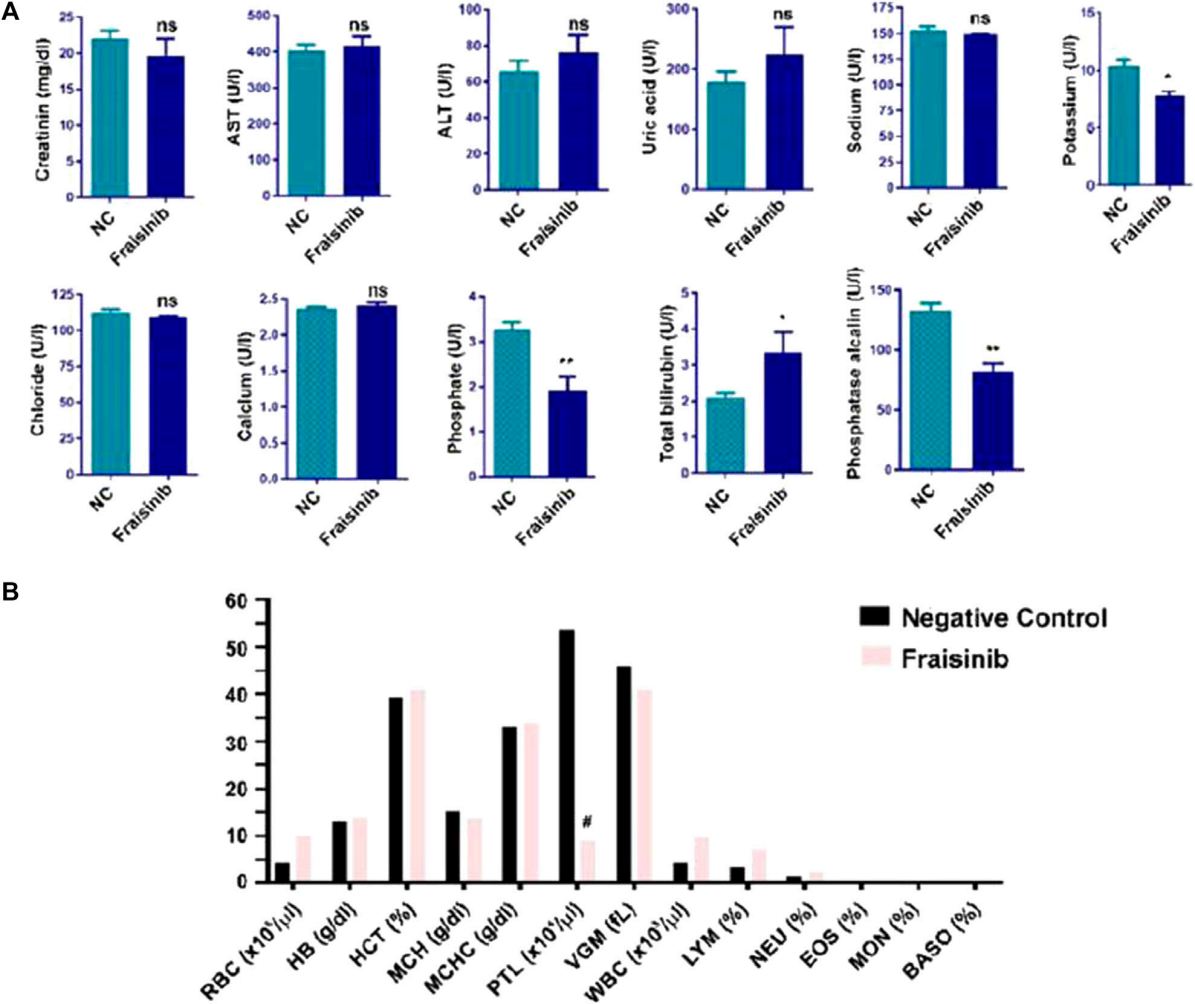
Hematological and biochemical analysis of mice blood from two groups of mice (each group n = 6), one treated with 125 mg/kg Fraisinib/BW and the other with vehicle (NC negative control). **(A)** Serum levels of creatinine; AST, aspartate aminotransferase; ALT, alanine aminotransferase; uric acid; sodium; potassium; chloride; calcium; phosphate; total bilirubin; and phosphatase alkaline. **(B)** Blood count results: RBC, red blood cells; HB, hemoglobin; HCT, hematocrit; MCH, mean corpuscular hemoglobin; MCHC, mean corpuscular hemoglobin concentration; PLT, platelet count; VGM, mean corpuscular volume; WBC, white blood cell; LYM, lymphocytes; NEU, neutrophils; EOS, eosinophils; MON, monocytes; and BASO, basophiles. The figures shown are mean ± standard error. #, values that differed significantly from the NC group. Statistical comparisons between groups were analyzed using a one-way analysis of variance (ANOVA). Statistical significance was considered for *p*-value <0.05. *p < 0.05; **p < 0.01. ns: not significant compared to the NC group.

In this work, to proceed further with the evaluation of toxicity, a group of six mice was treated for 30 days with a higher dose of 125 mg/kg BW three times a week and compared to a control group of six mice. Compared to the previous study, a wider range of physiological parameters was considered. Creatinine, AST, ALT, uric acid, sodium, potassium, chloride, calcium, and total bilirubin were within the norm, while phosphate and phosphatase alkaline were slightly lower, and total bilirubin was slightly higher than in the control group (Figure 3A). No significant changes in hematological and biochemical parameters were detected, and the blood count of RBC, HB, HCT, MCH, MCHC, VGM, WBC, LYM, NEU, EOS, MON, and BASO were in the norm, with PLT lower than in the control (Figure 3B). Overall, these results confirm that the drug should be well tolerated if used for cancer treatment.

### 3.4 Anti-tumor potential *in vivo*

**FIGURE 4:**
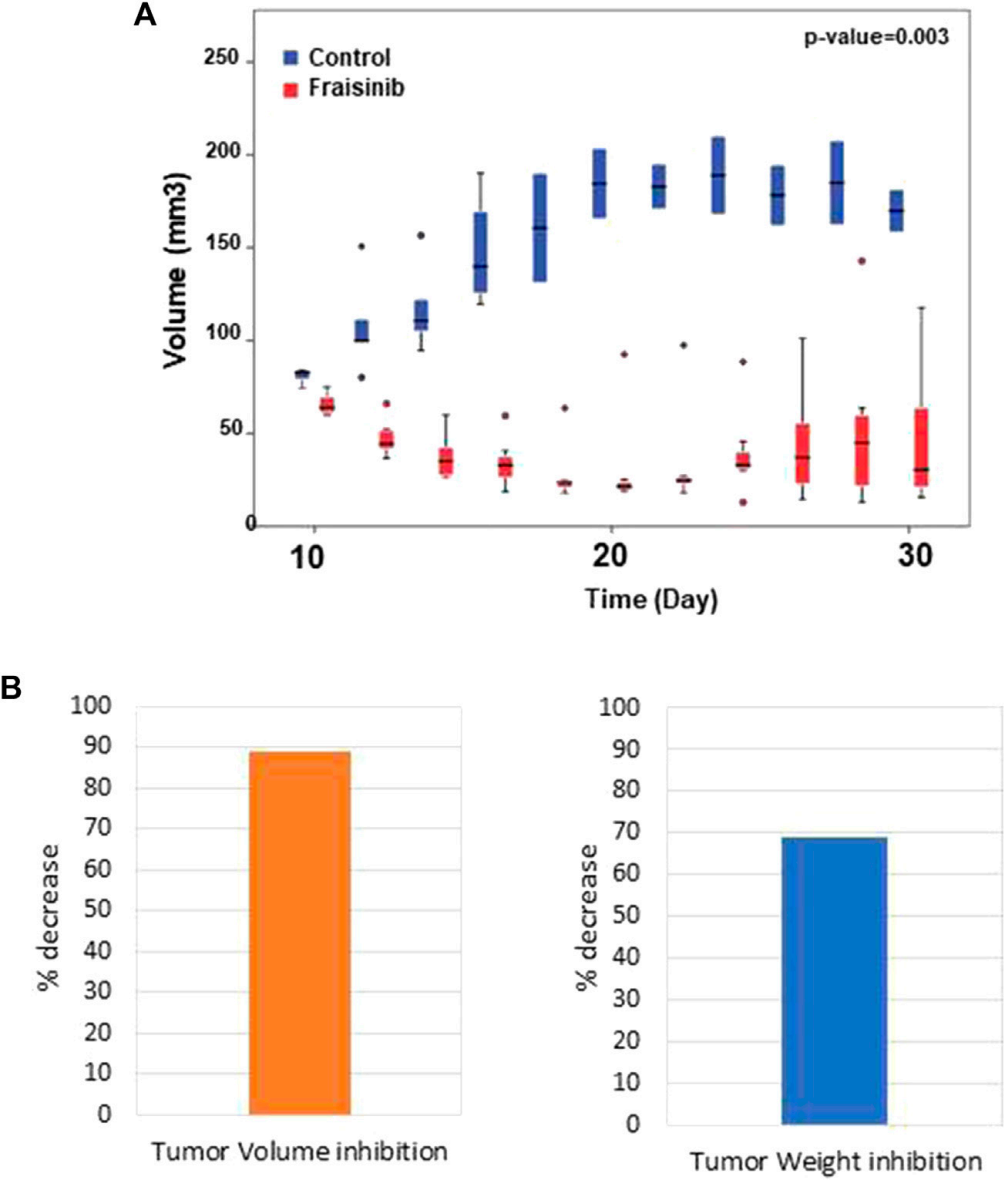
Inhibitory effect of Fraisinib treatment on tumor growth. **(A)** Box plot representing the tumor size of treated (red) and untreated (blue) animal groups at each time point. Circles represent the outliers. Unpaired *t*-test on the overall days' comparison between groups *p*-value = 0.003 **(B)** Tumor inhibition observed at the end of treatment compared to the untreated group: 89% and 69% volume and weight, respectively.

The anti-tumor effect of Fraisinib was evaluated *in vivo* in immunodeficient CD-1 nude mice (Crl:CD1-Foxn1nu). The tumor was considered to be developed 7 days after injection with the tumor cells. From that point in time, mice in the treatment group (n = 7) were administered a dose of 125 mg/kg BW of Fraisinib three times a week for 24 days. The control group (n = 5) received an equivalent volume of the vehicle mixture. Fraisinib treatment dramatically reduced the size and the weight of tumors from the first week of treatment compared to the control group (Figures 4A–B). Moreover, the data in Figure 4B showing the variation of tumor size as a percentage with respect to time 0 indicate that 10 days from the beginning of the treatment, the control group had a tumor size that was nearly nine-fold larger than in the treated group. Although the Fraisinib effect was evident, pairwise comparisons between the control and treatment groups at each time point did not reveal statistically significant differences, likely due to the low number of samples. This is due to the death of 3/5 control samples. Nevertheless, the statistical analysis of the tumor size in the overall groups over time showed a *p*-value of 0.03, thus suggesting that the medium values between the different groups were significantly different.

### 3.5 Target identification: *in silico* protein database screening and ranking

**FIGURE 5:**
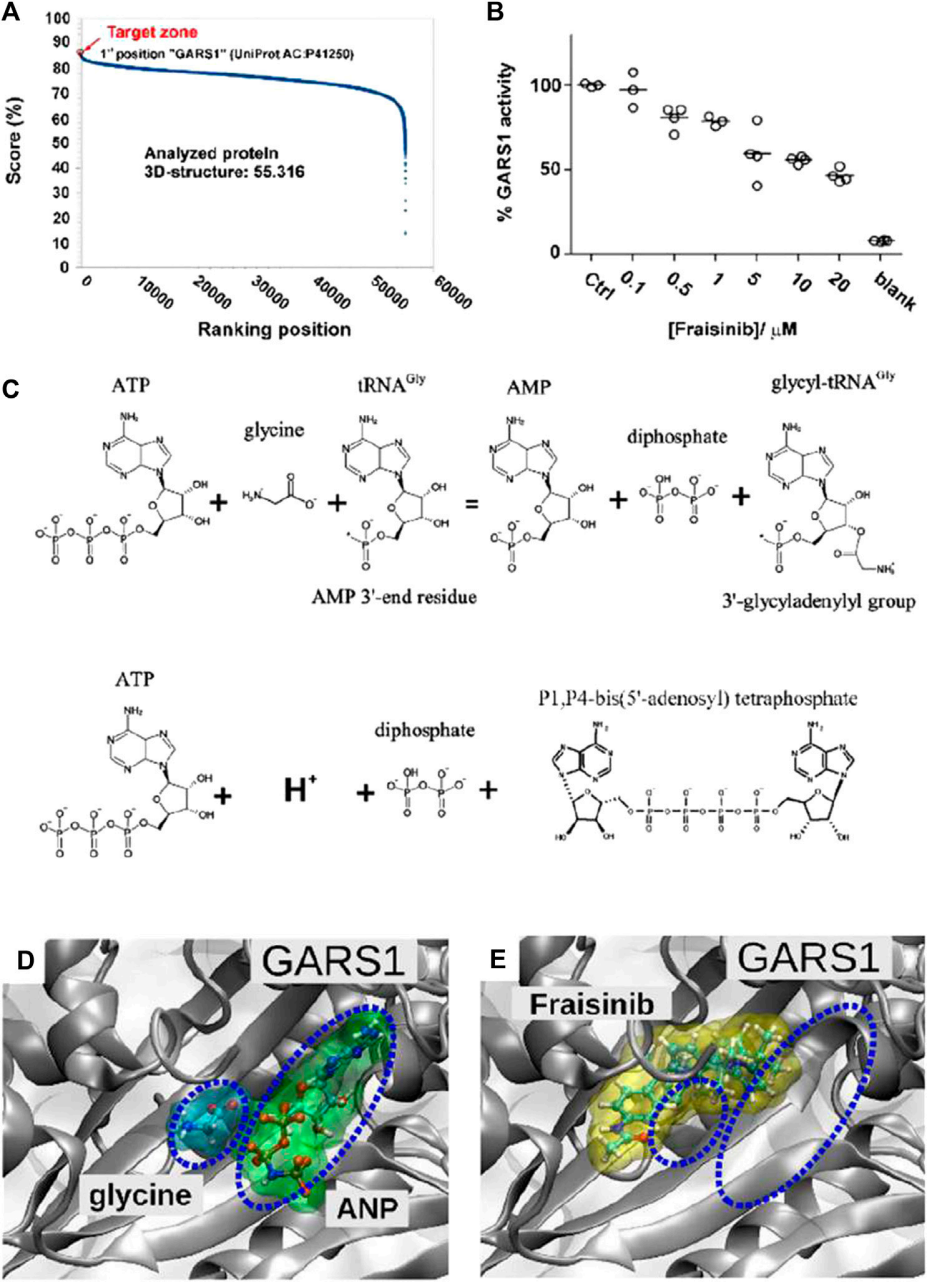
Target determination and validation. **(A)** SPILLO-PBSS screening and ranking for Fraisinib. The plot resulted from the *in silico* screening for Fraisinib on the available structural proteomes. Proteins are ranked in descending order of score. Glycyl-tRNA synthetase GARS1 (PDB code: 4KR3 - UniProtKB AC: P41250) was identified as the top-ranked potential target for Fraisinib. **(B)** Enzyme activity obtained by monitoring the amount of pyrophosphate (PPi) released during ATP consumption by GARS1 (circles) in the presence of increasing concentrations of inhibitor Fraisinib (0.1–20 µM). Enzyme activities are reported as average percentages (straight lines) normalized against enzyme activity measured in the absence of an inhibitor. **(C)** Two enzymatic reactions catalyzed by GARS1. **(D)** Positions of glycine and ATP (in this specific structure replaced by the nonhydrolyzable ATP analog 5′-adenylyl-imidodiphosphate (ANP)) and **(E)** Fraisinib within their corresponding binding sites obtained by X-ray diffraction (PDB code: 4KR3) and SPILLO-PBSS calculation, respectively. The partial overlap between the binding sites of glycine/ANP and Fraisinib is shown, which implies a competition between the various molecules for the same binding region, leading to the inhibition of the catalytic activity of the enzyme [drawings were produced using VMD (Götz et al., 2019)].

With the aim of identifying the potential target proteins of Fraisinib that could account for its anti-tumoral effect on the A549 cell line, the SPILLO-PBSS software was used (Di Domizio et al., 2014) to screen and rank a large protein database, including the available structural proteomes (Protein Data Bank 55,316 protein 3D structures, June 2020, see Materials and methods) of three different organisms, namely, *Homo sapiens*, *Mus musculus*, and *Rattus norvegicus*. This analysis generated the plot in Figure 5A, where points correspond to proteins ranked in descending order based on the greatest similarity between the reference binding site (RBS) and the best potential binding site (PBS) identified within each protein 3D structure. Interestingly, the non-linearity of the curve highlights the presence of a minority of proteins with scores higher than all the others, corresponding to the potential targets of Fraisinib.

### 3.6 GARS1 as a potential target for Fraisinib: competitive inhibition hypothesis

A potential binding site for Fraisinib was identified within the catalytic site of the enzyme, where the glycilation of tRNA in the presence of ATP takes place. Notably, GARS1 was identified by SPILLO-PBSS as a possible target of Fraisinib despite the presence of many steric clashes in the PBS, which would have prevented its detection by traditional structure-based methods. Importantly, the PBS turned out to partially overlap the region occupied by glycine and ATP, which in the reported structure was replaced by the isosteric ANP to improve the quality of diffraction data (Qin et al., 2014). We were, thus, able to hypothesize an inhibition of the catalytic activity of GARS1 by Fraisinib (Figures 5D, E) as the possible cause of the experimentally observed biological effects induced by Fraisinib. It cannot be excluded that the possible binding of Fraisinib may make GARS1 less flexible, interfering with its ability to interact with certain components of the neddylation pathway. Overall, these findings prompted us to conduct experiments to test the SPILLO-PBSS-predicted interaction between GARS1 and Fraisinib.

### 3.7 Effects of Fraisinib on the enzymatic activity of GARS1

Previous reports (Guo et al., 2009) showed that GARS1 catalyzes direct condensation of two ATP molecules to produce diadenosine tetraphosphate (Ap4a) and inorganic pyrophosphate (PPi) (Figure 5C). This reaction can easily be monitored by measuring PPi production by spectrophotometric assays (Upson et al., 1996; Grasso et al., 2015). The spectrophotometric assay was employed to investigate the inhibitory effect of Fraisinib against GARS1 activity. We observed that Fraisinib in the concentration range (0.1–20 µM) inhibited GARS1 activity dose-dependently (Figure 5B). In contrast, an isomer of Fraisinib (FHK563 in Supplementary Figure S2) and another four molecules mimicking diverse portions of this compound (Supplementary Figure S2A) were shown to be ineffective, further corroborating the hypothesis that Fraisinib is a specific and effective inhibitor of the GARS1 (Supplementary Figure S2B).

### 3.8 Evaluation of the biological role of GARS1 in the A549 cell line treated with Fraisinib

Adopting a multi-proteomic approach, the possible involvement of GARS1 in the regulation of phenotypic alterations that reduced *in vivo* tumorigenicity of A549 cells exposed to Fraisinib over time was evaluated. For this purpose, a differential proteomic analysis followed by the generation of functional networks and enrichment analysis was performed. Results obtained after including the known GARS1 interactors identified in the A549 cell line were subsequently compared with the previous one (Supplementary Figure S3). These results allowed for the identification of proteins deregulated by the treatment that are part of the interactome of the GARS1 protein.

In detail, a label-free differential proteomics analysis of the A549 cell line treated with 10 μM Fraisinib for 24, 48, and 72 h, compared to a DMSO control (Supplementary Table S1), was performed by mass spectrometry (MS). Among the 4,352 proteins identified, 1,076 (24.7%) were significantly dysregulated (*p*-value <0.05), with an expression change of more than two-fold. For each single time point analyzed, ∼11% of proteins were found altered; of these, 61.7% were downregulated after 24 h and approximately 50% were downregulated after 48 h and 72 h. Fifty-one proteins were altered at each of the three time points analyzed, while about 250 proteins were found altered at one of the time points (Supplementary Figure S4).

**FIGURE 6:**
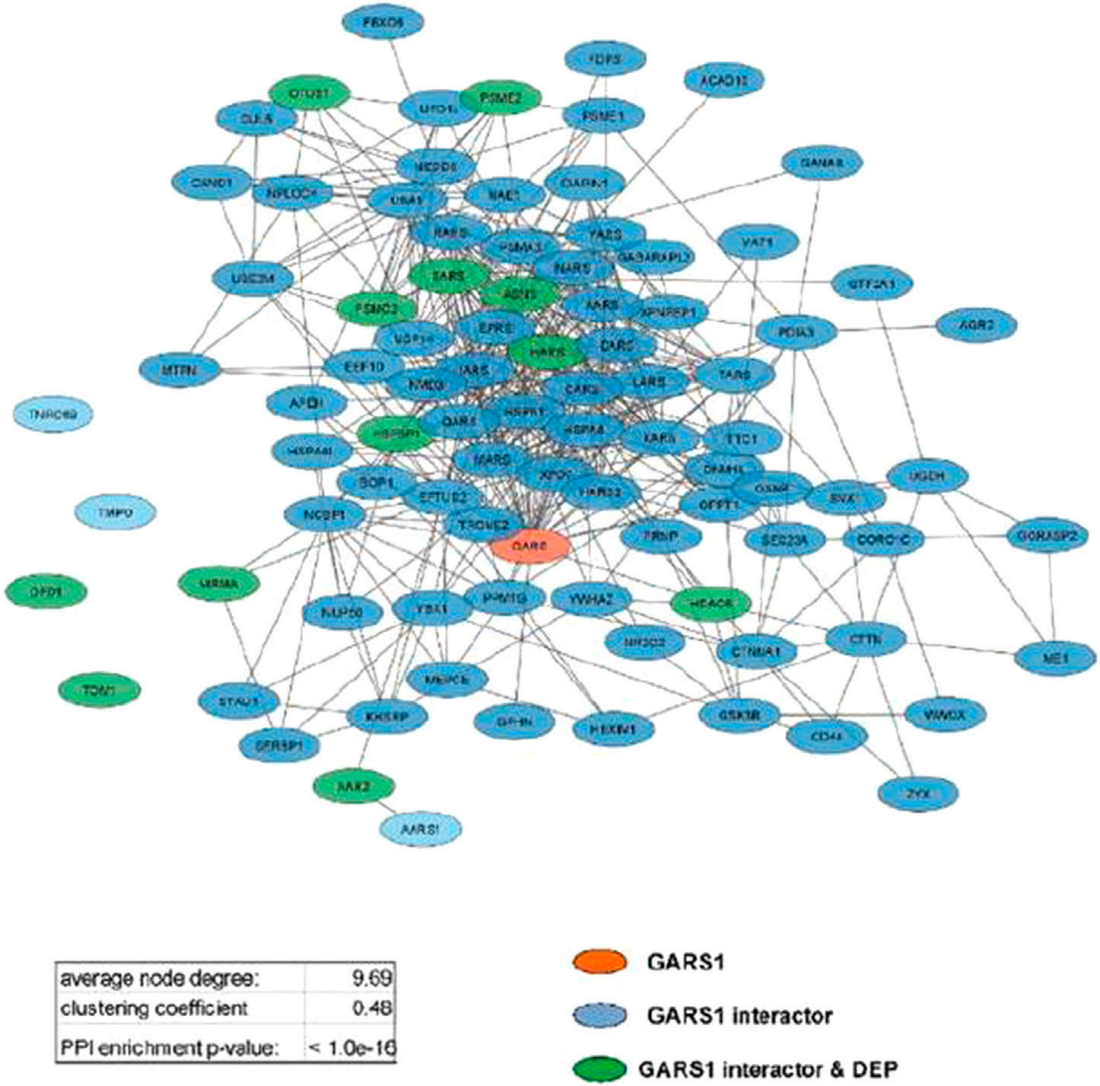
GARS1 interactome in the A549 cell line. Network parameters are reported in the inset table.

Analyzing protein interaction data obtained by the STRING database, three different functional networks were built using Cytoscape (Supplementary Figures S5A, C, E) to show the DEPs induced by Fraisinib treatment at 24, 48, and 72 h, hereafter defined as DEP24 h, DEP48 h, and DEP72 h. The DEP-network analysis results (see Table 1) revealed that ∼90% of DEP participated in network construction (node) with an average of ∼5 interactions among themselves (average number of neighbors), displaying a good density degree of connections (clustering coefficient ∼0.2) and a very low PPI enrichment *p*-value, indicating that DEP24 h, DEP48 h, and DEP72 h are indeed functionally and biologically connected groups of proteins. In network-based studies, there is increasing evidence demonstrating that it is critical to evaluate a set of genes/proteins based on their context-specificity expression, and it is also necessary to assess their neighbors even if these are non-significantly differentially expressed (Guan et al., 2012; Sonawane et al., 2017). As GARS1 expression was unchanged throughout Fraisinib treatment, we checked for the presence of its interactors in the DEP lists. For this purpose, from the STRING and PINA (Wu et al., 2009) databases, 140 proteins physically and functionally associated with GARS1 were retrieved, and from these, the 91 proteins (Supplementary Table S2) present in the protein list identified by MS were selected in order to build the GARS1 A549-specific interactome (Figure 6). Of note, 12 out of these 91 GARS1 interactors were also DEPs, suggesting that connections between DEP and GARS1 exist. This hypothesis was confirmed by building three merged networks integrating the three DEP lists and GARS1 interactors (Supplementary Figures S5B, D, F). Exploiting the resulting merged networks, newly participating nodes were found (Table 1) associated with a densely interconnected network where the central nodes were mainly GARS1 interactors and GARS1 itself. On measuring the topological features (Table 1), the thus obtained results, confirmed that the merged-network nodes tend to cluster together more efficiently than in DEP networks, indicating a crosstalk between GARS1 interactors and DEPs at all time points of treatment.

**FIGURE 7:**
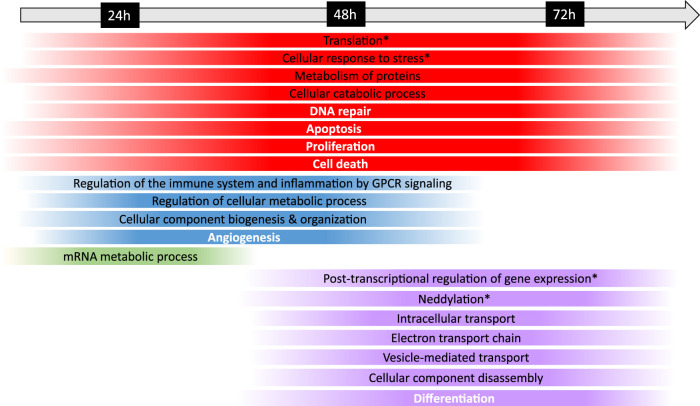
Timeline of the biological mechanisms dysregulated in the A549 cells following Fraisinib treatment. The chronological progression of pathway dysregulation, observed by comparing the DMSO control and Fraisinib-treated cells at 24, 48, and 72 h, is shown over time. Under the timeline, each strip spans the first and last treatment time point at which the biological mechanism resulted dysregulated; the strips are color coded as follows: red for biological mechanisms altered at all time points; blue at 24 and 48 h, green only at 24 h, and violet at 48 and 72 h. The colors at the edges of each strip are faded as the data were obtained at definite time points and both the start and the end of each process cannot be exactly defined. General biological mechanisms are written in black, and cancer hallmarks are written in bold white; the biological mechanisms specifically regulated by GARS1 are indicated with an asterisk (retrieved from Cancer Gene Net, https://signor.uniroma2.it/CancerGeneNet/).

In addition, to further evaluate the biological mechanisms underlying Fraisinib treatment, the enrichment analysis of the 91 GARS1 interactors and the DEP24 h, DEP48 h, and DEP72 h was performed using the Cytoscape ClueGO plugin. The results obtained for GARS1 interactors revealed its involvement in regulating not only tRNA amino-acetylation, as expected, but also other cellular functions related to protein and mRNA metabolism (Supplementary Figure S6). Comparing the ClueGO network results linked to the analysis of the three DEP lists with or without GARS1 interactors (Supplementary Tables S1, S2; Supplementary Figures S11–S13) showed an evident improvement in enriched terms for the latter due to the addition of GARS1 interactors to DEP lists (bubble graphs in Supplementary Figures S7–S9), allowing a better characterization of the cellular and functional mechanisms that were dysregulated during treatment with Fraisinib (Figure 7).

At the time points of 24, 48, and 72 h, the dysregulation of distinct pathways mediated by the interactions of GARS1 (Supplementary Table S3) and its neighborhood (i.e., DEP24 h, DEP48 h, and DEP72 h) was observed, and the results are in line with other studies reporting the dependency of ARS functions on cellular expression patterns (Lee et al., 2006; Kwon et al., 2019; Wang and Yang, 2020; Sung et al., 2022).

In particular, the timeline showed a prompt response of mRNA processing at 24 h, leading to post-transcriptional regulation processes occurring over a longer time span, as expected from the succession of events involved in mRNA maturation and functioning (Alpert et al., 2017).

At 24 and 48 h, molecular profiling of the response to Fraisinib treatment showed dysregulated processes related to cellular metabolism, immune response, inflammation, and angiogenesis. At 48 and 72 h, other biological mechanisms involved in the control of cellular fate, such as differentiation and gene expression, showed dysregulation (Figure 7). Interestingly, at these time points, enrichment analysis highlighted neddylation, an important biological process controlling protein function that is involved in cellular activity (Mo et al., 2016) and lung cancer tumorigenesis (Li et al., 2014).

## 4 Discussion

The modern drug discovery process is still full of hurdles and is becoming increasingly harder every year. Finding a biological system that has not previously been assessed as a therapeutic target is an extraordinary challenge despite the growing number of scientists involved in the field and the large number of novel discoveries. In this context, our group has pioneered the application in the medicinal chemistry of calix[n]pyrroles. Although this class of molecules has been known for over a century (Baeyer, 1886), it is only recently that we proposed their use as possible drug carriers (Cafeo et al., 2013) and, later, as potential drugs (Lappano et al., 2015). More recently, a macrocyclic compound called Fraisinib [*meso*-(p-acetamidophenyl)-calix (Sève and Dumontet, 2005) pyrrole] that can successfully kill cancer cells while inducing very limited (or no) toxicity (Geretto et al., 2018; Ben Toumia et al., 2022) has been synthesized and tested *in vitro*, *ex vivo*, and *in vivo*. The present study demonstrates how Fraisinib is able to drastically reduce the volume and weight of NSCLC tumors within the first week of treatment without inducing severe toxicity in mice (Figures 4A, B). To provide a possible biomolecular interpretation of the experimentally observed biological effects induced by Fraisinib in the human lung carcinoma A549 cell line, we performed a 3D *in silico* screening on a proteome-wide scale using SPILLO-PBSS software. As subsequently confirmed experimentally, the software succeeded in identifying an inhibitory interaction between Fraisinib and the catalytic site of the GARS1 enzyme, which was predicted as the top-ranked target of Fraisinib out of more than 55,000 protein structures analyzed (Figure 5A). Fraisinib should, therefore, be considered a “first-in-class” compound that can inhibit GARS1 enzymatic functions and, hence, also regulate Ap4A levels in the cells.

NSCLC treatment options and recommendations depend on several factors, including the type and the stage of cancer, the possible side effects induced by the drug(s), and the patient’s overall health. The therapeutic options for this tumor include surgery, radiation therapy, chemotherapy, targeted therapy, and immunotherapy. Additionally, chemo-treatments can be administered before and after surgery to lower the risk of tumor recurrence and to help reduce the extent of surgery.

In particular, chemotherapies are based on the use of cytotoxic compounds, and they are based on a regimen that usually consists of a specific number of cycles given over a set period of time. The most popular drugs available for NSCLC are Carboplatin, Cisplatin, Taxotere, Etoposide, and Taxol (Sève and Dumontet, 2005; Derks et al., 2017). These drugs may also damage healthy cells in the body, thus causing unpalatable side effects.

Targeted therapies are focused on blocking specific proteins that are essential to cancer growth and survival, and often, targeted therapies are administered together with conventional chemotherapeutics such as platinum-based drugs. The novel drug reported here to be effective on cancer cell growth could be inserted in the category of target therapy due to the identification of GARS1 as its specific molecular target and, similar to other treatments, we cannot exclude the possibility that it could be suitable to be used in combination with conventional approaches.

In particular, the identification of the Fraisinib target GARS1 is absolutely in accordance with the role of GARS1 in cancerogenesis. As a consequence of GARS1 inhibition, Fraisinib can modulate different biological and molecular pathways related to GARS1 functions in a unique way. The glycyl-tRNA synthetase GARS1 belongs to the aminoacyl-tRNA synthetases (ARSs) enzyme family that plays essential roles in cells, catalyzing the aminoacylation of tRNA substrates by juxtaposing ATP, amino acids, and tRNAs (Figure 5C), and the produced aminoacylated tRNAs are used in protein synthesis by the ribosomes (Cader et al., 2007). In the absence of its aminoacid cognate, glycine, GARS1 produces the metabolite diadenosine tetraphosphate (Ap4A) by the direct condensation of two ATP moieties, releasing pyrophosphate (Guo et al., 2009). Ap4A is produced in response to various environmental and genotoxic stresses. Although its biological role has still not been fully elucidated, Ap4A is reported to possibly be involved in different signaling pathways (Ferguson et al., 2020).

GARS1 emerges prominently in all analyses as the aminoacyl-tRNA synthetase (aaRS) most strongly associated with cancer. This prominence may be attributed to its dual role in facilitating protein synthesis in both the cytosol and mitochondria. In contrast to other ARSs, GARS1 predominantly exhibits gene amplification in various cancer types, while other proteins tend to feature more deletions and mutations rather than amplifications. Furthermore, higher levels of GARS1 mRNA are associated with significantly poorer survival rates in different cancer types, among which is lung adenocarcinoma, thus confirming the pathogenic role of this protein in cancer development.

Notably, prior research has uncovered multiple seemingly unrelated functions for GARS1 beyond its conventional role in protein synthesis. GARS1 appears to play a critical role in the neddylation pathway (Wang et al., 2011), which facilitates the attachment of the ubiquitin-like protein NEDD8 to specific protein substrates (Mo et al., 2016). NEDD8 is a ubiquitin-like protein that participates in post-translational protein modification, a process referred to as neddylation. Neddylation not only controls ubiquitination modifications but also influences a range of biological processes, thus playing a crucial role in the onset and prognosis of lung cancer. In particular, the inhibition of the neddylation pathway exhibited a potent anti-proliferative effect on platinum drug‐resistant A549 and H460 cells, and the clinical investigation of protein neddylation inhibition was proposed as a novel strategy for the treatment of Pt‐resistant NSCLC (Jazaeri et al., 2013).

Resistance to platinum drugs leads to cancer recurrence and the failure of the therapeutic plan treatment and, therefore, a poor prognosis. The upregulation of the NEDD8-binding enzyme UBE2F is a significant pathway for lung cancer cells to evade platinum-induced apoptosis. Following platinum-based drug treatment, UBE2F, as a substrate, exhibits reduced binding capacity to CUL3, leading to the accumulation of UBE2F. However, the accumulation of UBE2F, in conjunction with RBX2, promotes the neddylation of CUL5, subsequently facilitating the degradation of the substrate NOXA. This results in reduced cellular oxidative stress resistance and diminished cell survival. These findings also suggest that UBE2F could serve as a promising new therapeutic target. From the proteomics analysis on A549 cells, Fraisinib has been shown to be a potential inhibitor of neddylation; therefore, it could, thereby, indirectly suppress UBE2F activity and allow NOXA to further promote apoptosis.

Studies have suggested that GARS1 modulates the cell cycle through its involvement in neddylation, raising the possibility that targeting GARS1 could inhibit cancer, as exemplified by small molecule neddylation inhibitors. Furthermore, bovine GARS1 has been found to enhance mTOR activation by translocating to the nucleus in response to amino acid signaling (Yu et al., 2019). While this activity has yet to be definitively demonstrated in humans, the conservation of the localization signal in human GARS1 implies that mTOR activation through GARS1 could be an advantageous pathway exploited by cancer cells.

Contrary to these previous findings, secreted GARS1 has also been observed to induce apoptosis in tumor cells by binding to K-cadherin on the cell surface and releasing phosphatase 2A (PP2A), resulting in ERK dephosphorylation and apoptosis (Park et al., 2012).

It is noteworthy that there exists a strong correlation between glycine consumption and the expression of the mitochondrial glycine biosynthesis pathway across various cancer cells, suggesting that GARS1’s involvement in cancer may also relate to glycine metabolism. Presently, it remains unclear which of these activities prevails in the context of cancer or whether there is a discernible pattern of tissue specificity among them. Nonetheless, our analyses indicate that GARS1 represents a promising target for multiple cancer types, possibly owing to one or more of these functional roles.

These findings pointed us toward GARS1 as an important factor in the Fraisinib-induced biological effect observed in A549 cells.

Growing evidence supports the role of ARSs in sustaining cancer phenotype and, consequently, makes them promising targets for cancer therapies (Wang and Yang, 2020; Sangha and Kantidakis, 2022; Sung et al., 2022). Multi-omics analysis, evaluating genetic alteration and prognostic value of transcriptional dysregulation, shows GARS1 to have the highest association with cancer out of all the ARSs (Wang et al., 2020), especially for lung adenocarcinoma (Zhang et al., 2021). GARS1 mRNA overexpression, found at the tissue level, has recently been linked to poor prognosis for hepatocellular carcinoma (Wang et al., 2022) and was selected as a urinary protein marker for the diagnosis of urothelial carcinoma (Chen et al., 2021). Although the enzymatic activity of GARS1 is principally involved in the first essential step of protein synthesis, other functions associated with cancer evolution and cellular homeostasis have been described in the last decade (Sung et al., 2022).

Data from differential proteomics and bioinformatics analyses unraveled that Fraisinib, affecting the catalytic activity of GARS1, can modulate different biological and molecular functions involved in antagonizing the tumoral phenotype of NSCLC A549 cells over time. Evaluating the effects of Fraisinib treatment in A549 cells over time, protein metabolism, the cellular catabolic process, and four pathways involved in sustaining the cancerous phenotype (proliferation, apoptosis, DNA repair, and cell death) were altered throughout the treatment (Figure 7; Supplementary Table S4). The relationship between the alterations of these cellular processes and cancer formation mechanisms described for other ARSs (Lee et al., 2006; Wang and Yang, 2020; Sung et al., 2022) indicates that Fraisinib’s anti-tumoral activity could depend on GARS1 targeting. In addition, the timeline representing the effects of the drug is consistent with the progression of biological processes, starting from pathways involved in protein expression immediately modulated by the molecule toward more complex processes involving vesicle-mediated pathways such as intracellular trafficking, which are modulated later.

Of note, 145 and 68 DEPs involved in cell cycle and apoptosis regulation, respectively, were found to be modulated by Fraisinib in agreement with the results obtained in cell-based assays, thus allowing the mode of action of the molecule to be better defined and its biological target to be identified. It was also demonstrated that Fraisinib can inhibit the invasive ability of A549 cells. The pathogenetic role of GARS1 in oncology has recently been confirmed by a publication reporting that the silencing of GARS1 expression is effective in counteracting the progression of prostate cancer (Khosh Kish et al., 2023). These data, together with the proven ability of this compound to cross the blood–brain barrier (BBB) (Geretto et al., 2018), suggest that Fraisinib can kill two birds with one stone: targeting the primary tumor and its metastases “in one shot.” Taken together, this work suggests that the inhibition of GARS1 expression and/or GARS1 enzymatic activity may be innovative molecular targets for cancer treatment.

## 5 Conclusion

In this work, we report the beneficial effects of the molecule Fraisinib in counteracting lung tumors in both the A549 human adenocarcinoma cells and an NSCLC mouse model xenografted with this cell line. In addition, the identification of GARS1 as the drug target and the analysis of pathways deregulated by Fraisinib open new perspectives in the search for additional therapeutic adjuvants for this severe cancer.

## Data Availability

The datasets presented in this study can be found in online repositories. The names of the repository/repositories and accession number(s) can be found at: https://www.ebi.ac.uk/pride/archive/, PXD037653.
