# Supplementary material for: Fraisinib: a calixpyrrole derivative reducing A549 cell-derived NSCLC tumor in vivo acts as a ligand of the glycine-tRNA synthase, a new molecular target in oncology
Source: Front Pharmacol. 2024 Jan 3;14:1258108. doi: 10.3389/fphar.2023.1258108 (PMC10791888; doi:10.3389/fphar.2023.1258108)
Supplement: Supplementary file 2 [file Image1.PDF]

Supplementary Information

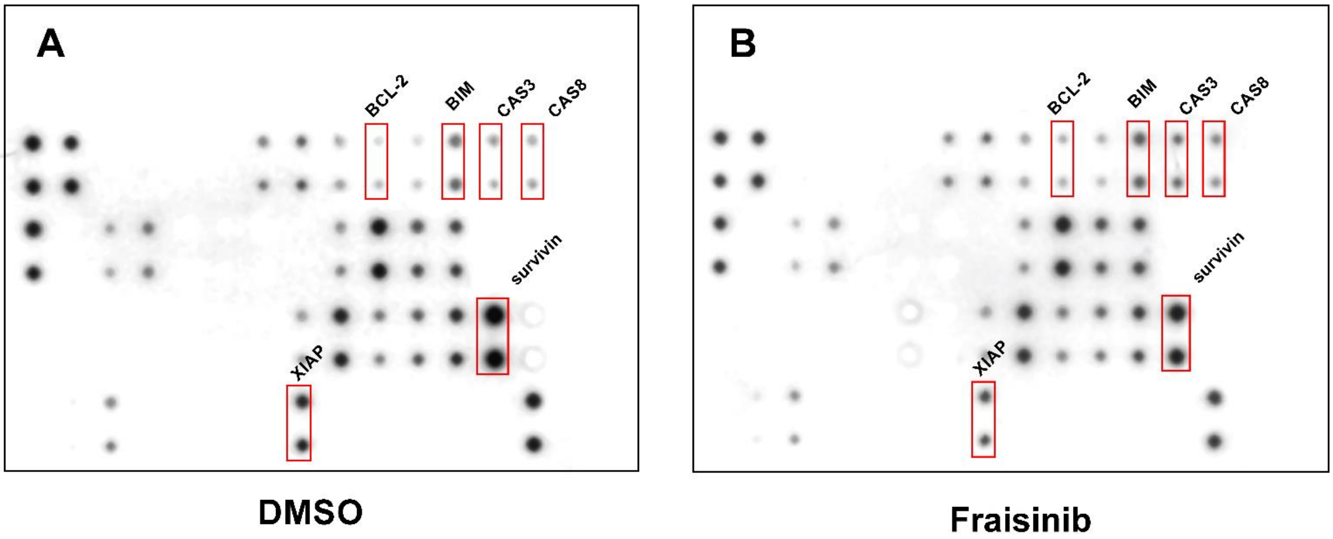

Fig. S1: Human apoptotic proteins array for (A) control (0.01% DMSO) A549 cells and (B) cells treated at concentration of 10 μM with Fraisinib

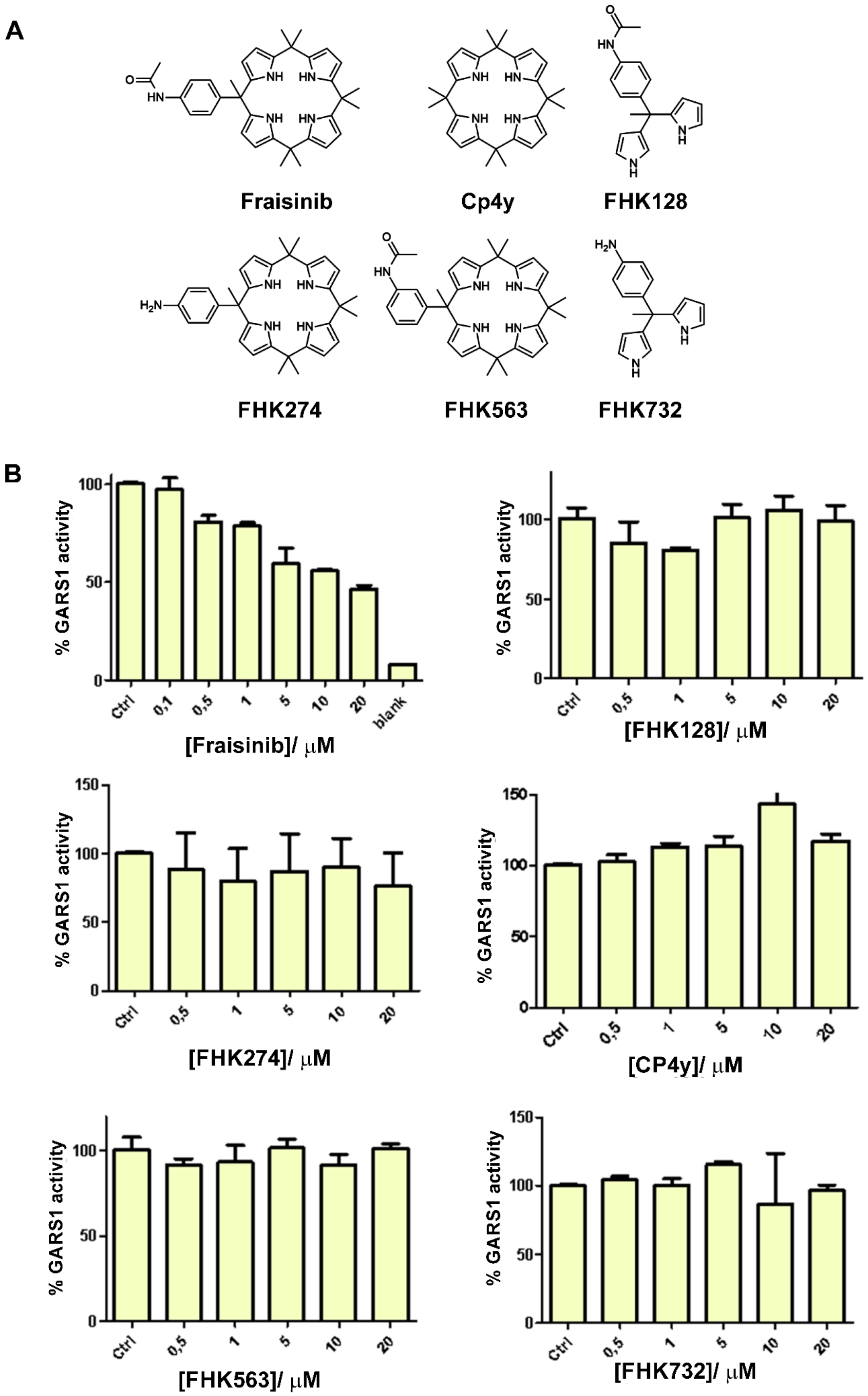

Fig. S2: GARS1 inhibition by Fraisinib and different macrocyclic compounds. A) Formulae of the tested compounds B) GARS1 Activity inhibition at different concentrations

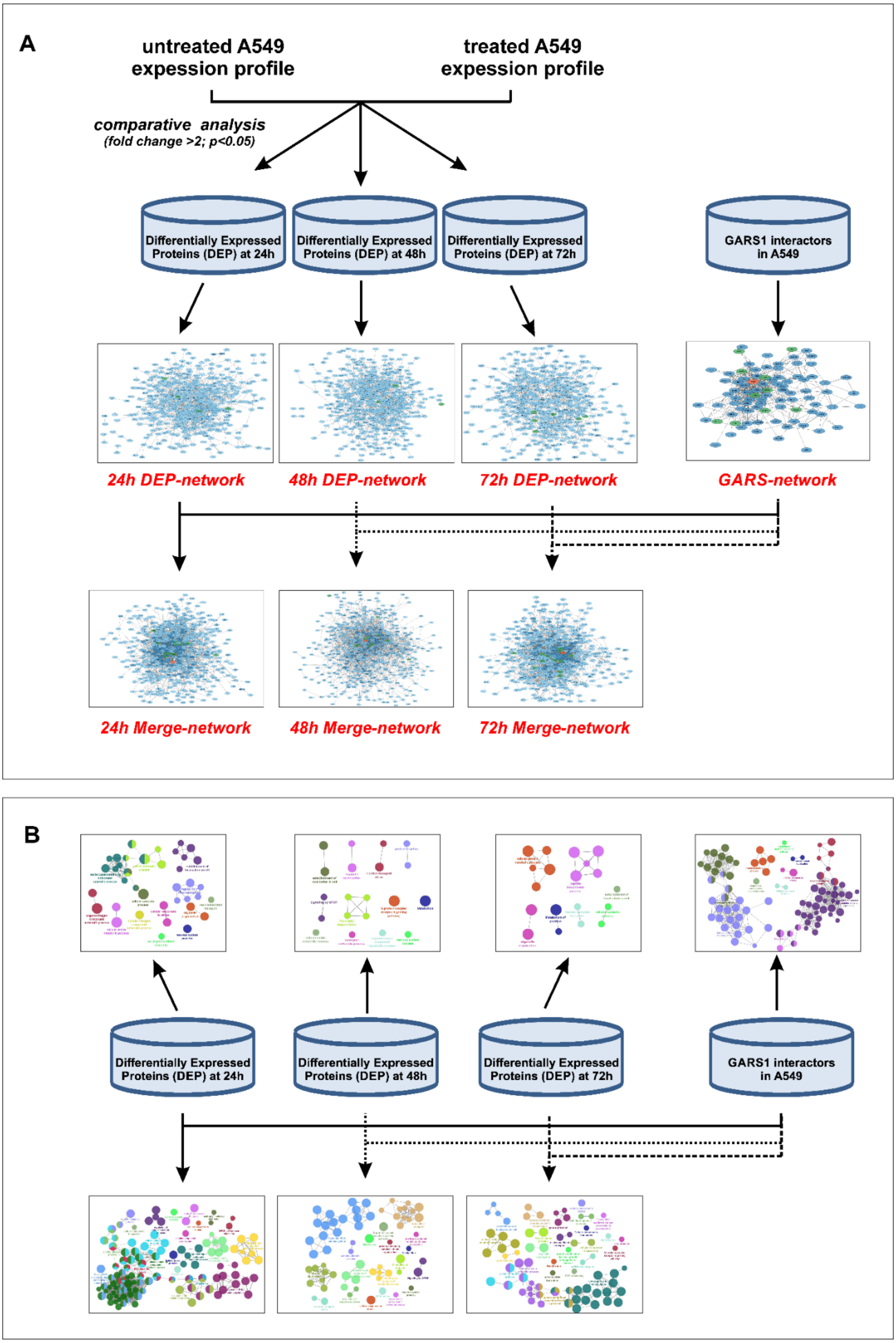

Fig. S3. Workflow graph of proteomic and bioinformatic analyses used in this study. A) Differential proteomic analysis and functional network construction by Cytoscape using protein-protein interaction data retrieved from STRING. B) Enrichment analysis workflow to build ClueGO-networks using Cytoscape plugin.

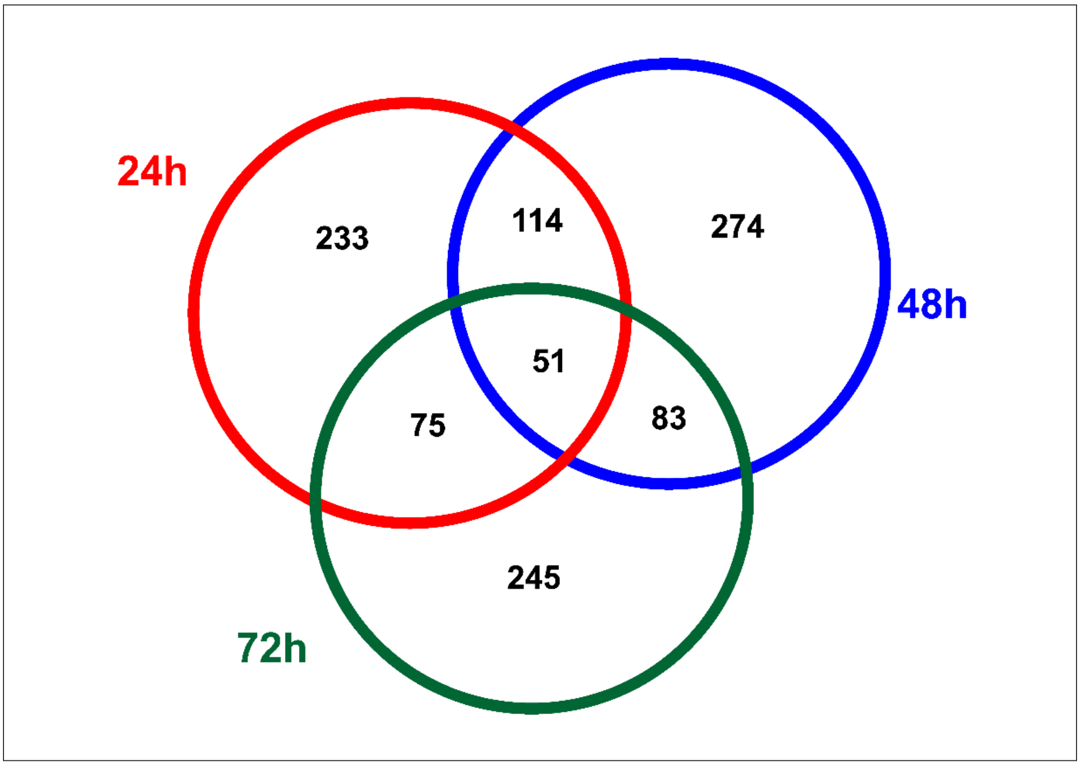

Treatment time (h)	DEP number (% of total)	Number of UP-regulated (% of total DEP)	Number of DOWN-regulated (% of total DEP)
24	473 (10.9)	181 (38.2)	292 (61.7)
48	522 (12)	260 (49.8)	262 (50.2)
72	454 (10.4)	221 (48.7)	233 (51.3)
DEP: differentially expressed proteins			

Fig. S4. Comparative analysis results. For each time point, MS data of Fraisinib treated vs untreated A549 cell line were evaluated by Proteome Discoverer software. The number of proteins and the percentage with respect to the total amount (in parenthesis) has been reported.

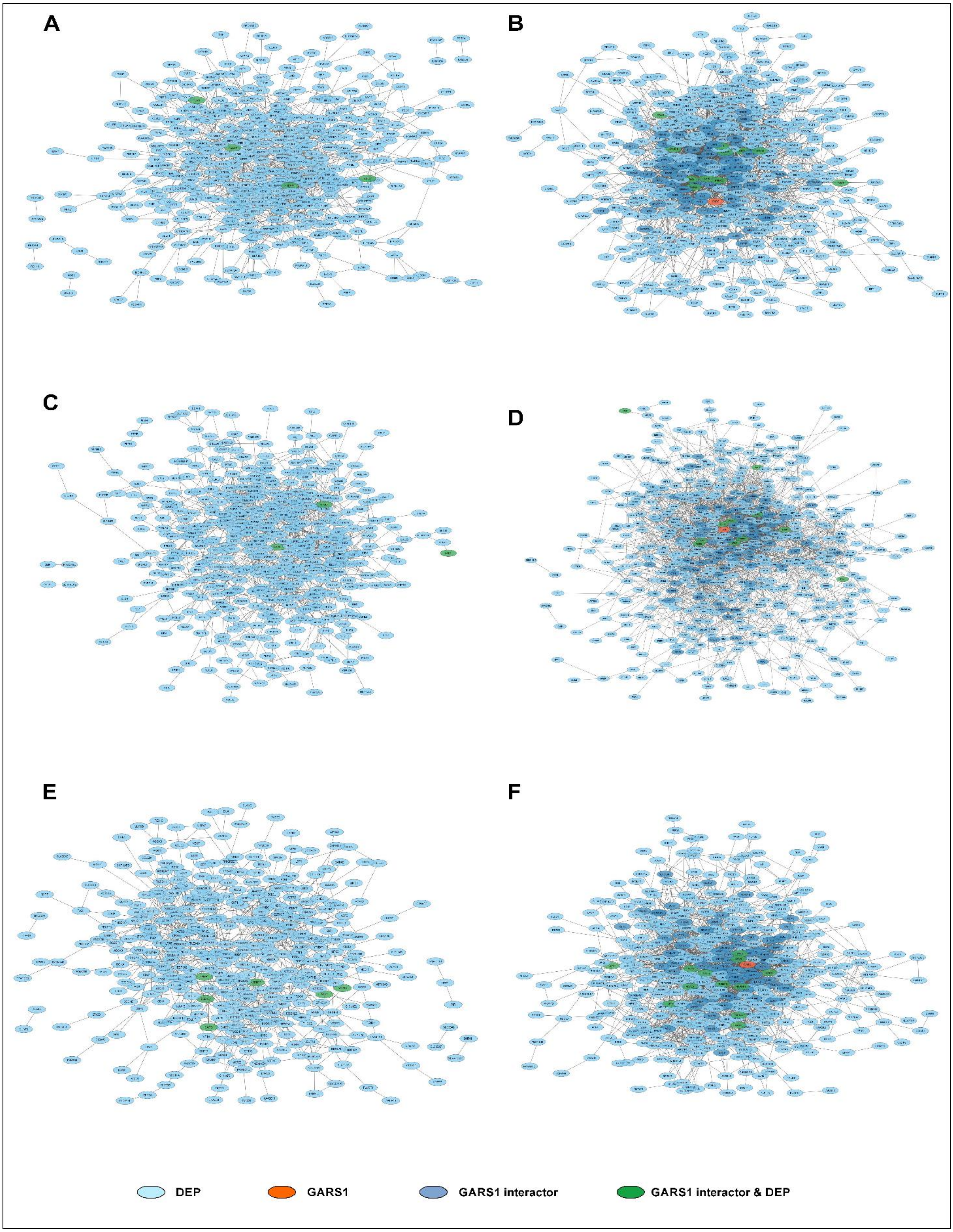

Fig. S5. Comparison between protein-protein interaction networks of DEP without (A,C,E) or with GARS1interactors (B,D,E) obtained from A549 cell cultured for 24h (A,B), 48h (C,D) and 72h (E,F). The networks parameters are reported in Table 1. The disconnected nodes were hidden.

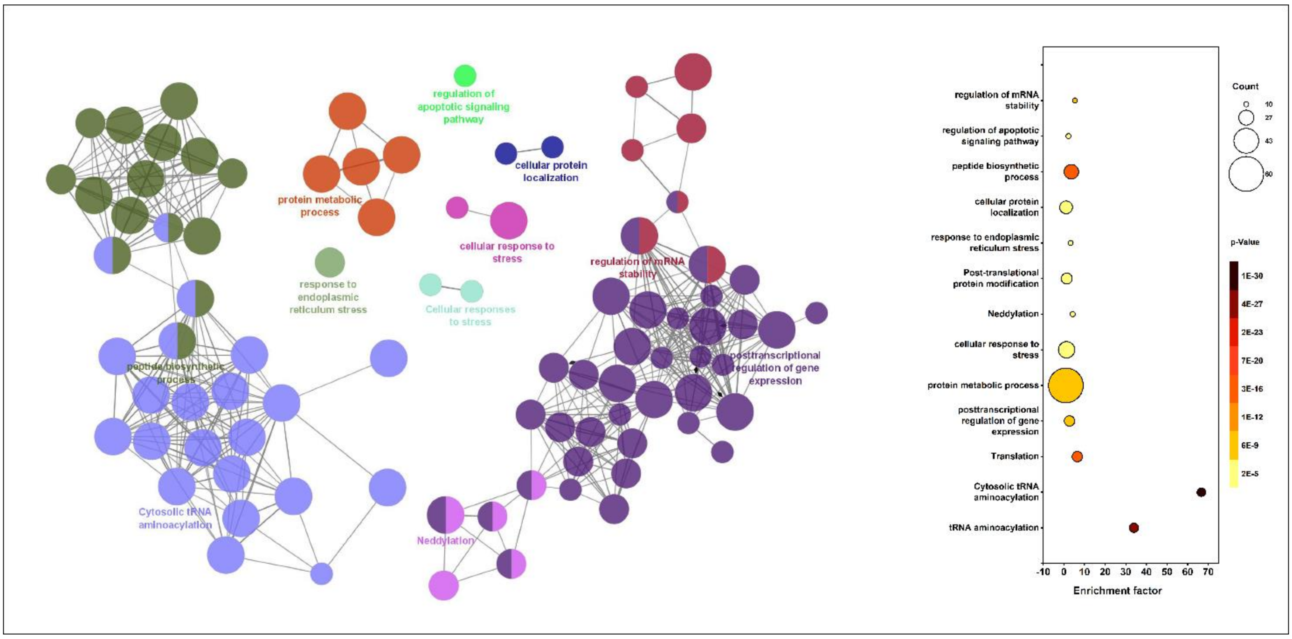

Fig. S6. Graphical overview of ClueGO analysis of the 91 GARS1 interactors. Bubble graph on the right shows the enrichment score of terms/pathways reported in the network. The circle size and the color referred to number of enriched proteins and the p-value, respectively. The p-value significance degree, corrected with Bonferroni step down, is shown as color scale on the right.

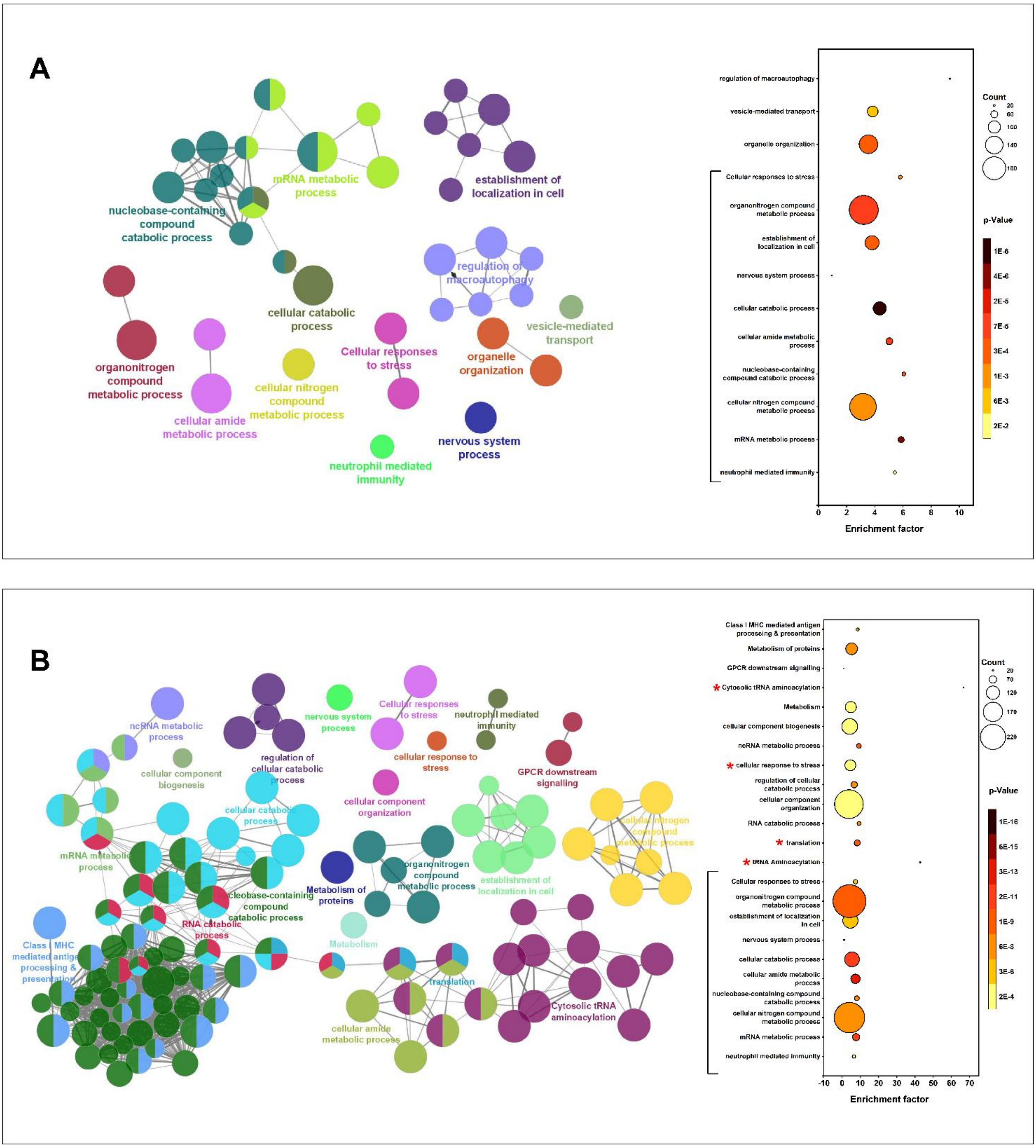

Fig. S7. Graphical overview of ClueGO analysis of the 24h DEP without (A) or with (B) the 91 GARS1 interactors. Bubble graph on the right shows the enrichment score of terms/pathways reported in the network. The circle size and the color referred to number of enriched proteins and the p-value, respectively. The p-value significance degree, corrected with Bonferroni step down, is shown as color scale on the right. Bracket in A and B highlights the same term/pathway. Red asterisk indicates term/pathway common with GlueGO analysis of GARS1 interactors in fig S6.

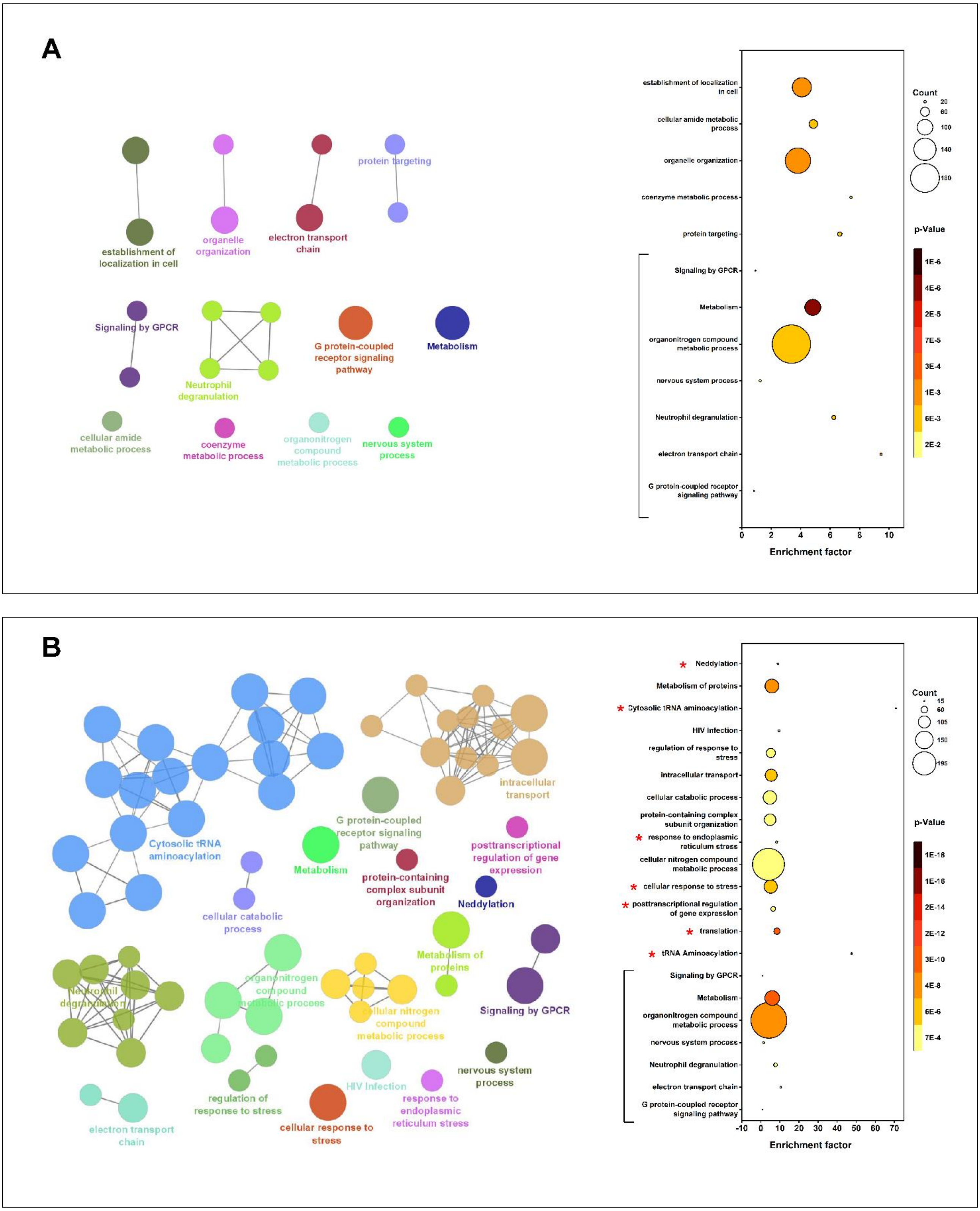

Fig. S8. Graphical overview of ClueGO analysis of 48h DEP without (A) or with (B) the 91 GARS1 interactors. Bubble graph on the right shows the enrichment score of terms/pathways reported in the network. The circle size and the color referred to number of enriched proteins and the p-value, respectively. The p-value significance degree, corrected with Bonferroni step down, is shown as color

scale on the right. Bracket in A and B highlights the same term/pathway. Red asterisk indicates term/pathway common with GlueGO analysis of GARS1 interactors in fig S6.

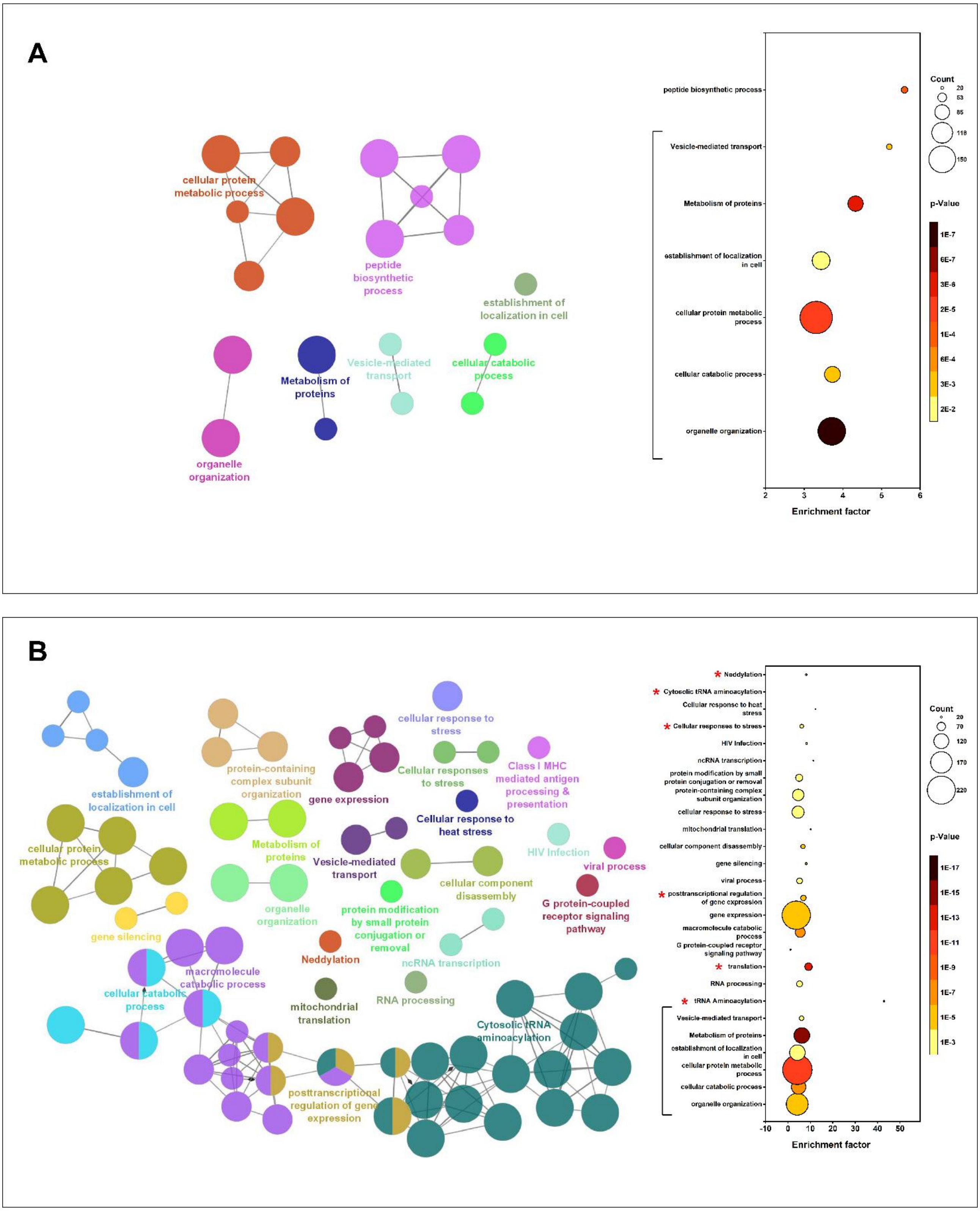

Fig. S9. Graphical overview of ClueGO analysis of 72h DEP without (A) or with (B) the 91 GARS1 interactors. Bubble graph on the right shows the enrichment score of terms/pathways reported in the network. The circle size and the color referred to number of enriched proteins and the p-value,

respectively. The p-value significance degree, corrected with Bonferroni step down, is shown as color scale on the right. Bracket in A and B highlighted the same term/pathway. Red asterisk indicates term/pathway common with GlueGO analysis of GARS1 interactors in fig S6.

Supplementary References

SR1. Parodi F, Carosio R, Ragusa M, et al. Epigenetic dysregulation in neuroblastoma: A tale of miRNAs and DNA methylation. Biochim Biophys Acta. 2016;1859(12):1502-1514.

SR2. Ferrari N, Granata I, Capaia M, et al. Adaptive phenotype drives resistance to androgen deprivation therapy in prostate cancer. Cell Commun Signal. 2017;15(1):51.
